# Female‐specific resource limitation does not make the opportunity for selection more female biased

**DOI:** 10.1111/evo.14106

**Published:** 2020-10-20

**Authors:** Ivain Martinossi‐Allibert, Johanna Liljestrand Rönn, Elina Immonen

**Affiliations:** ^1^ Department of Organismal Biology/Systematics Biology Uppsala University Uppsala SE‐75236 Sweden; ^2^ Department of Ecology and Genetics/Animal Ecology Uppsala University Uppsala SE‐75236 Sweden; ^3^ Department of Ecology and Genetics/Evolutionary Biology Uppsala University Uppsala SE‐75236 Sweden

**Keywords:** Laboratory settings, opportunity for selection, sex bias, sex‐specific selection, sexual dimorphism, sexual selection

## Abstract

Competition for limiting resources and stress can magnify variance in fitness and therefore selection. But even in a common environment, the strength of selection can differ across the sexes, as their fitness is often limited by different factors. Indeed, most taxa show stronger selection in males, a bias often ascribed to intense competition for access to mating partners. This sex bias could reverberate on many aspects of evolution, from speed of adaptation to genome evolution. It is unclear, however, whether stronger opportunity for selection in males is a pattern robust to sex‐specific stress or resource limitation. We test this in the model species *Callosobruchus maculatus* by comparing female and male opportunity for selection (i) with and without limitation of quality oviposition sites, and (ii) under delayed age at oviposition. Decreasing the abundance of the resource key to females or increasing their reproductive age was challenging, as shown by a reduction in mean fitness, but opportunity for selection remained stronger in males across all treatments, and even more so when oviposition sites were limiting. This suggests that males remain the more variable sex independent of context, and that the opportunity for selection through males is indirectly affected by female‐specific resource limitation.

Variation in fitness among individuals is what natural selection acts on. It can be partitioned into variation among individuals in their genetic makeup (breeding value), in their phenotypic condition subject to environmental variation, and to the interaction between the two (Arnold and Wade 1984). Therefore, the extent to which genetic variation will be translated into variation in fitness visible to natural selection depends on context, through the availability of key developmental resources and the intensity of competition among individuals (Hoffmann and Hercus 2000; Zikovitz and Agrawal 2013). For example, under abundant resources individual variation in resource acquisition should matter little to fitness, but if resources are scarce even slight differences in acquisition traits may translate into large differences in fitness.

To see how variation in fitness translates into opportunity for selection, it is useful to think about a selection differential, which is the covariance between a trait and relative fitness (Robertson 1966; Arnold and Wade 1984). Variance in relative fitness then sets the upper limit for the strength of selection on any trait (Crow 1958), as it represents the strength of selection on a trait that would covary perfectly with fitness. For this reason, variance in relative fitness has been called the opportunity for selection, often designated by *I*. When a change in context affects the magnitude of fitness differences among individuals, it affects variance in fitness, and thus the opportunity for selection.

In sexually reproducing populations, males and females often have different reproductive strategies (Andersson 1994), which means that they can be limited by different resources. This results in a situation where a common environment can impose different challenges to the sexes, which should translate into sex‐specific variance in fitness (equivalent to sex‐specific opportunity for selection). Indeed, sexual selection theory predicts that mating partners should often be a limiting resource for males, which together with natural selection should result in generally stronger net selection on males than on females (Wade and Arnold 1980). If so, this could have far‐reaching consequences in several aspects of evolutionary biology, such as speed of purging of deleterious mutations (Whitlock and Agrawal 2009), speed of adaptation to novel environments (Martinossi‐Allibert et al. 2019), rate of evolution of sex‐specific traits, or genome structure and evolution (Wright and Mank 2013). For example, the purging of deleterious mutations through selection on males, at the benefit of both sexes, has been proposed as one of the mechanism explaining the maintenance of sexual reproduction itself (Siller 2001; Whitlock and Agrawal 2009). Differences across the sexes in fitness variance can also lead to different effective population sizes in the sexes, which can cause asymmetries in the genetic diversity of the sex chromosomes relative to autosomes (Caballero 1995; Charlesworth 2001; Charlesworth 2009).

Empirical studies investigating patterns of sex‐specific selection show that in many species variance in reproductive success is indeed greater in males (e.g., Clutton‐Brock 1988; McLain 1991; Fleming and Gross 1994; Webster et al. 2001; Setchell et al. 2005), but not in all (reviewed in Snyder and Gowaty 2007). In their recent meta‐analysis, Janicke et al. (2016) gathered sex‐specific estimates of variance in reproductive success and other selection metrics from 66 species in 72 studies. Their work showed that although there is variation across taxa and some species show stronger selection in females or no sex bias, the general trend is for stronger selection in males (as measured with greater variance in reproductive success in males). In 2018, Singh and Punzalan (2018) collated data from sex‐specific estimates of phenotypic selection on traits (selection gradients) in wild populations. With 865 estimates, they detected generally stronger selection in males, mostly driven by traits related to mating success. These two comprehensive studies therefore clearly support the hypothesis that there should be generally stronger selection in males, with some evidence indicating that this trend may be due to sexual selection specifically. However, these two studies have also revealed tremendous variability across taxa, and the source of this variability is still poorly understood.

If male fitness is expected to generally be more variable because of sexual selection, there are also many reasons for female fitness to exhibit high levels of variance. First, in some species they do experience strong sexual selection (reviewed in Hare and Simmons 2018), but there are also many other sources of fitness variation depending on the ecology of each species, such as competition for nutritional resources, nesting, or oviposition sites (Stockley and Bro‐Jørgensen 2011). The context in which selection is measured greatly matters, as the limitation of specific resources can magnify or shrink fitness differences among individuals. Because the sexes are sensitive to different limiting resources, variation in environmental conditions could unveil variation in fitness differently in the sexes, which has rarely been experimentally studied (but see Zikovitz and Agrawal 2013). Here, we tested this hypothesis, and thus robustness of the pattern of greater opportunity for selection in males, by measuring sex‐specific variance in relative fitness using three experimental conditions designed to specifically challenge female fitness. Using individual‐based simulations, we predicted that female‐specific stress or resource limitation should result in more female‐biased opportunity for selection. We tested this prediction in a population of the seed beetle *Callosobruchus maculatus*, a widely used laboratory system for sexual selection studies (Zuk et al. 2014). We compared sex‐specific variance in relative fitness (the opportunity for selection) in three different treatments: under a competitive context allowing sexual competition on both sexes and ad libitum oviposition substrate offered to females (control treatment [CT]), under a heterogeneous environment treatment (HT), presenting individuals with the context of sexual competition but with an oviposition substrate of heterogeneous quality; and an ageing treatment (AT) in which females were challenged physiologically to delay their age at oviposition. This last treatment was chosen to challenge individuals through ageing, which is known to affect the sexes differently in *C. maculatus* (Fox et al. 2003; Maklakov and Arnqvist 2009; Immonen et al. 2016). For example, eggs from older mothers are less likely to hatch, whereas there are no detectable effects of paternal age on offspring phenotype (Fox et al. 2003). Fertility also declines much more rapidly with age in females than in males (Immonen et al. 2016). Ageing therefore represents a greater challenge for female fitness in this species, which may result in more female‐biased opportunity for selection. Moreover, both the HT and AT should be relevant to the ecology of *C. maculatus* that, as a bean beetle, is dependent on irregular seed availability as the only larval food resource, without which the females do not even lay eggs. Females have evolved a great capacity to detect a high‐quality bean resource as their oviposition site (Mitchell 1975; Cope and Fox 2003). The HT thus provides a challenge that can reveal variation in this ability crucial for female fitness, whereas the AT represents a situation faced by individuals required to postpone reproduction in the absence of available bean resources. We estimated the opportunity for selection as: *I* = *σ*_w_^2^/*ѿ* ^2^, where *σ*_w_ is the standard deviation in fitness and *ѿ* the mean fitness (Crow 1958).

Individual‐based simulations, relying on the assumption that stress increases variance in individual condition, predicted that sex‐specific stress should make the opportunity for selection more biased toward the more stressed sex, even if intrasexual competition for mating is biased toward one sex. For example, even if only males compete with each other while females do not, increasing female‐specific stress results in a more female‐biased opportunity for selection.

Empirical results, however, showed that the opportunity for selection was consistently higher in males than in females, and the male‐bias was even stronger under oviposition site limitation (i.e., HT). Mean fitness, measured as the number of adult offspring recruited to the next generation, was lower in both HT and AT compared to the control, indicating they were generally challenging conditions. These results, put in perspective with our individual‐based simulations, suggest that stronger selection in males may after all be robust to changes in sex‐specific selection, possibly because male variation can be indirectly affected through interaction with females. Understanding better female‐specific environmental limitations should further our understanding of the natural variation in sex‐specific patterns of selection.

## Methods

### INDIVIDUAL‐BASED SIMULATIONS

#### Overview

We developed individual‐based simulations to generate predictions regarding sex‐specific opportunity for selection under stress. First, a population of males and females was simulated by drawing individuals from a distribution of condition values. Environmental stress was then modeled on each sex independently. Each individual was then attributed a fitness value according to a model of sexual reproduction, and finally the opportunity for selection was calculated from the fitness of all individuals in the population. The model of sexual reproduction takes into account condition‐dependent female fecundity and condition‐dependent male mating success in competition. We describe the details of this basic form of the simulations below. We also explored effects of additional features of sexual reproduction, such as female competition, sperm limitation, male harm, and nuptial gifts, which we present in Supporting Information S1.

#### Individual condition

The condition of each individual is drawn from a normal distribution of mean 1. The mean is always fixed to one, because the condition is relative within each sex. The standard deviation of the normal distribution varies according to the level of stress.

#### Simulation of sex‐specific stress

Stress is simulated by increasing the standard deviation of the distribution of conditions. This represents the expression of cryptic genetic variation revealed by new or stressful environments. The standard deviation of the distribution of conditions can be varied independently for each sex (Fig. S1).

#### Sexual reproduction

To mimic closely the experimental design that the present simulation makes predictions for, we consider sexual reproduction in groups of four individuals, two of each sex. The two females are defined by their conditions, noted *c*_f1_ and *c*_f2_ for each female, respectively. The two males are also defined by their conditions noted *c*_m1_ and *c*_m2_.

#### Female fitness as a function of female condition

Each female is able to produce a number of fertile eggs, which depends on her condition *c*_f1_. We assume here, for the sake of simplicity, that female competition and potentially harmful or beneficial interactions with males during mating (nuptial gifts, male harm) do not occur, but we relax these assumptions and expand the model accordingly in Supporting Information S1. Here, we simply assume that female fecundity scales linearly with female condition:

$$\mathrm{Eggs}\;=c_{f1}.$$

Finally, the fitness of the focal female is the proportion of these fertile eggs that get fertilized by males. We assume here that sperm is never limiting so that female fitness is equal to female fecundity; in Supporting Information S1, we also relax that assumption and explore the role of sperm limitation.

#### Male fitness as a function of male condition and female fecundity

In each group of four individuals, the two males share the total fecundity of the two females, as calculated by the female fitness function presented above. Males compete for their portion of fecundity, following the function

$$\mathrm{Male}\;\mathrm{fitness}\;=\;\mathrm{Total}\;\mathrm{female}\;\mathrm{fitness}\times \frac{{c_{m1}}^{g_{m}}}{{c_{m1}}^{g_{m}}+{c_{m2}}^{g_{m}}},$$

where *c*_m1_ and *c*_m2_ are the conditions of the focal and competitor males, respectively, and $g_{m}$ is the parameter representing the intensity of male‐male competition.

#### Running the individual‐based simulations

We use the software Mathematica (version 11.0, Wolfram 2016) to sample individual condition and implement the sexual reproduction model, as well as for production of graphical output. The Mathematica code used to produce the simulation results of this manuscript as well as the ones of Supporting Information S1 can be found in Supporting Information S2. For each simulation run, a population size of 1000 is assumed.

### STUDY ORGANISM AND POPULATION

The seed beetle *C. maculatus* is a facultative aphagous pest species found in grain storages and fields across West Africa and Asia. Its reproductive cycle, which typically spans over about a month, starts by adults laying eggs on the surface of beans (e.g., the black‐eyed bean *Vigna ungulata* used in the present study), after which larvae burrow and develop inside the beans until they emerge as reproductively mature adults.

The study population originates from a natural population sampled in Lome, Togo (06°10#N 01°13#E) in 2010. It has been kept under laboratory conditions since then (29°C, 12:12 light cycle, 50% humidity) with a constant population size of approximately 400–500 individuals. Fitness assays were also performed under laboratory conditions (29°C, 12:12 light cycle, 50% humidity).

### EXPERIMENTAL DESIGN

#### Fitness assays

Fitness was measured in lifetime competitive assays where one focal individual was placed together with a competitor of the same sex and two mating partners of the opposite sex inside a 9‐cm Petri dish. The environment inside the dish varied according to the treatment (see experimental treatments below). At the start of the experiment, all individuals were adult virgins collected less than 24 h after emergence from the beans. The competitor individual was sterilized by gamma radiation (100 Gy), a commonly used method in seed beetles that allows the competitor individual to compete for matings and achieve fertilizations, but insures that zygotes fertilized by the competitor will not develop due to the high number of double‐stranded breaks in the embryo DNA caused by the irradiation (Eady 1991; Maklakov et al. 2009). The four individuals were left to interact during their lifetime and offspring were counted as emerged adults of the next generation. A female fitness assay included one focal female, one sterilized female, and two male partners. The same design was used for the male fitness assays, which included one focal male, one sterilized male, and two female partners.

#### Experimental treatments

Our study included three treatments, aimed to create different reproductive challenges for the sexes.

The CT represents the laboratory setting classically used in *C. maculatus* studies: a 9‐cm Petri dish with ad libitum black‐eyed beans (27 g, approximately 130 beans). Although male fitness variation can be manifested through pre‐ and postmating sexual competition, for females this environment likely represents less challenges. Their oviposition substrate, the bean, is directly available, in a high and consistent quality, and in nonlimiting quantity. In this treatment, the fitness of 102 males and 102 females was measured.

The HT was designed to directly challenge females in their ability to discriminate the quality of oviposition sites. Each Petri dish was filled with beans of variable quality: 15 high‐quality beans (3–4 g) and the remainder of poor quality for a total of 27 g, as in the CT. The low‐quality beans were produced by letting a stock population of *C. maculatus* use the beans for larval development, resulting in bored beans that provide less resources for offspring to develop on. In this treatment, the fitness of 105 males and 87 females was measured.

The AT was designed to challenge females in their ability to withhold their reproduction until a suitable oviposition site is available. This treatment bears ecological relevance to a scenario where high‐quality oviposition sites are exhausted upon female hatching, requiring prolonged periods of searching for suitable sites. In this treatment, the four individuals were first placed in an empty dish and left to interact for 48 h, after which ad libitum (27 g) high‐quality beans were added. In this treatment, the fitness of 101 males and 97 females was measured.

For each treatment, the fitness of approximately 100 individuals of each sex was measured, for a total sample size of 594 fitness assays.

### STATISTICS

#### Mean fitness

The effect of experimental treatments on mean fitness (offspring number) was analyzed using a linear mixed model, as implemented in the lme4 package (version 1.1‐18‐1; Bates et al. 2015) in R (version 3.5.1; R Core Team 2018), taking into account normal distribution of the data. Experimental treatment, sex of the focal individual, and their interaction were specified as fixed effect and date of the fitness assay as a random effect.

#### Individual offspring weight

The effect of experimental treatments on individual offspring weight was analyzed using a linear mixed model, as implemented in the lme4 package in R, taking into account normal distribution of the data. Experimental treatment, sex of the focal individual, and their interaction were specified as fixed effect and the date of the fitness assay as a random effect.

#### Sex‐specific variance in fitness

A Bayesian model, as implemented in the MCMCglmm package in R (version 2.26; Hadfield 2010), was used to estimate components of variance in fitness attributed to each sex by experimental treatment combination. Because opportunity for selection is the variance in relative fitness, fitness data were mean standardized so that each sex by treatment subset had a mean of one prior to this analysis. The model was then specified with assay date as a random effect and the total phenotypic variance was estimated for each sex by experimental treatment combination (*idh* structure not allowing for covariances to be estimated). For each experimental treatment, the log ratio of the posterior distributions for male and female variances was then computed using posterior distributions from the model, giving a mean log ratio and 95% confidence intervals.

## Results

### INDIVIDUAL‐BASED SIMULATIONS

We present simulation results for the scenario where we assume that only males compete with each other, and neither beneficial or harmful interactions nor sperm limitation occurs during mating. This scenario matches well with what we know of our model system (strong male‐male competition) and remains relatively simple. Male harm and nuptial gifts are likely to occur as well in our study system but we examine those in Supporting Information S1 and show that they do not change qualitatively the effect of sex‐limited stress. We also assume here that females do not compete, which may not reflect accurately the situation of our experimental design but is a conservative assumption regarding our prediction, as we also show in Supporting Information S1.

**Figure 1:**
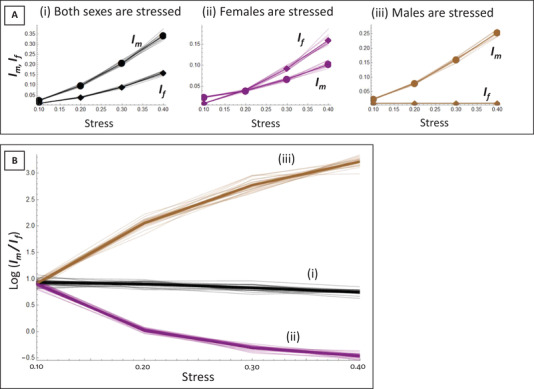
Simulated opportunity for selection under stress, sex‐specific (A) and logged sex ratio (B). Opportunity for selection is male biased in the case with no stress, because of intense male‐male competition (*g*_m_ = 2). Sex‐specific graphs (A) represent the opportunity for selection in females (*I*_f_) and males (*I*_m_) averaged over 10 simulations per sex and stress scenario. The logged sex‐ratio graph (B) represents the average of 20 simulations per line. On each graph, the “Stress” axis gives the value of the standard deviation of the distribution of individual condition in the population, around a fixed mean of 1.

In Figure 1, we show the effect of stress on sex‐specific opportunity for selection. We can see that in this scenario, when no stress is applied yet, opportunity for selection is higher in males. This is due to the parametrization of the model with intense male‐male competition (*g_m_* *= 2*). When stress is applied to both sexes, opportunity for selection increases faster in males. When stress is applied to females only, opportunity for selection increases in both sexes but faster in females. When stress is applied to males only, opportunity for selection increases in males but not in females. As a result, the sex ratio of opportunity for selection remains relatively consistent, and therefore male biased, when stress is applied to both sexes, but becomes more female biased when stress is limited to females and more male biased when stress is limited to males (see Fig. 1B). The outcome is not symmetrical in the two sex‐limited stress scenarios as male‐limited stress increases the male bias in selection in a more pronounced manner than the female‐limited stress increases the female bias in selection. This result is expected because the present scenario implies that stress acting on male condition will affect female fitness only little. Nevertheless, it is important to note that in spite of the strong male‐male competition and the absence of female‐female competition, female‐specific stress pushes opportunity for selection toward stronger selection in females.

### MEAN FITNESS

**Figure 2:**
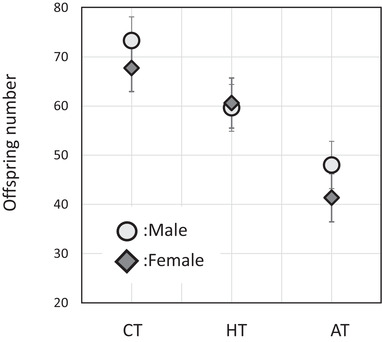
Mean fitness for each sex and experimental treatment. Mean fitness (adult offspring number) and 95% confidence limits (linear mixed model estimates) are given for each treatment: control (CT), heterogeneous treatment (HT), and ageing treatment (AT). Female values are given by dark‐shaded diamonds and male values by light‐shaded circles.

Mean fitness (offspring number) differed among all experimental treatments (Table 1), being highest in the CT followed by the HT and finally the AT (Fig. 2). The treatment differences from each other were confirmed by post hoc tests (Tukey's post hoc, CT‐AT: HSD = 8.6, *P* < 0.001; CT‐HT: HSD = 2.3, *P* = 0.024; AT‐HT: HSD = 6.0, *P* < 0.001; corrected for multiple testing with the Holm‐Bonferroni method). A weak main effect of sex was also detected (Table 1; Fig. 2) with male assays showing slightly overall higher mean offspring number but there was no sex by treatment interaction. These result indicate that the HT and AT were indeed challenging, with, respectively, 14% and 36% reduction in mean fitness compared to the control, and that the AT was more stressful than the HT.

**Table 1:** ANOVA table for a linear mixed model with offspring number as a response variable. Type III test. Date of the fitness assay was estimated as random effect

Effect	Chi square	df	*P*‐value
Intercept	275	1	<0.001
Treatment	78.4	2	<0.001
Sex	4.67	1	0.03
Treatment by sex	3.53	2	0.17

### TOTAL AND INDIVIDUAL OFFSPRING WEIGHT

**Figure 3:**
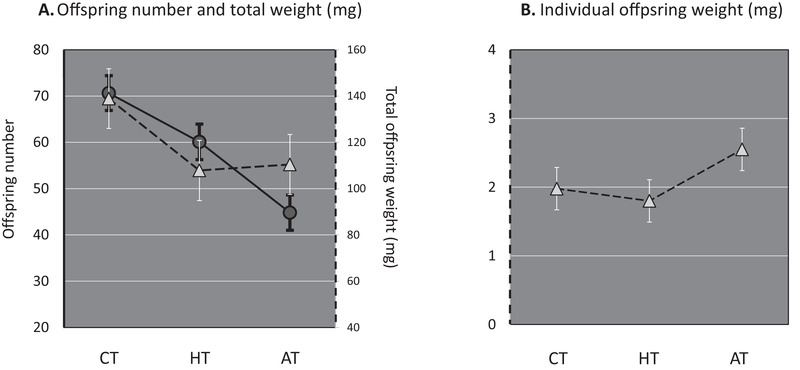
Mean fitness, total offspring weight, and individual offspring weight for each treatment. Mean fitness (adult offspring number, circles), mean total offspring weight (triangles, A), mean individual offspring weight (triangles, B), and 95% confidence intervals (linear mixed model estimates) are given for each treatment: control (CT), heterogeneous treatment (HT), and ageing treatment (AT).

Mean total offspring weight differed among the experimental treatments (Table 2): the CT had the highest total weight, whereas the HT and AT showed no difference (Fig. 3A; Tukey's post hoc: CT‐AT: HSD = 3.5, *P* = 0.001; CT‐HT: HSD = 2.9, *P* = 0.009; AT‐HT: HSD = 0.55, *P* = 0.58; corrected for multiple testing with the Holm‐Bonferroni method). Thus, the HT and AT had a different mean number of offspring, but the same mean total offspring weight. This is achieved by individuals from the AT producing larger offspring (Fig. 3B). More particularly, individual offspring weight was higher in the AT compared to both other treatments that did not differ from each other (Table 3; Tukey's post hoc: CT‐AT: HSD = 7.6, *P* < 0.001; CT‐HT: HSD = 1.8, *P* = 0.07; AT‐HT: HSD = 9.2, *P* < 0.001; corrected for multiple testing with the Holm‐Bonferroni method).

**Table 2:** ANOVA table for a linear mixed model with total offspring weight as a response variable. Type III test. Date of the fitness assay was estimated as random effect

Effect	Chi square	df	*P*‐value
Intercept	188	1	<0.001
Treatment	14.1	2	<0.001
Sex	2.49	1	0.11
Treatment by sex	3.02	2	0.22

**Table 3:** ANOVA table for a linear mixed model with individual offspring weight as a response variable. Type III test. Date of the fitness assay was estimated as random effect

Effect	Chi square	df	*P*‐value
Intercept	260	1	<0.001
Treatment	96.2	2	<0.001
Sex	3.89	1	0.049
Treatment by sex	3.12	2	0.21

### SEX‐SPECIFIC VARIANCE IN FITNESS

**Figure 4:**
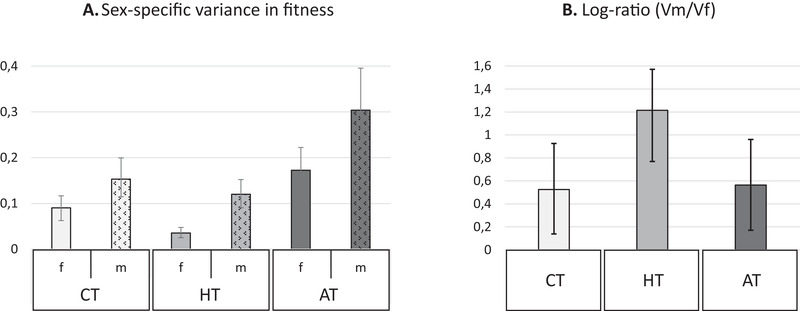
Sex‐specific variance in fitness and log ratio of male over female variance for each treatment. Estimates and 95% confidence interval (Bayesian model estimates) are given for (A) sex‐specific variance in fitness for females (f, empty bars) and males (m, patterned bars) and for (B) log ratio of male over female variance log (*V*_m_/*V*_f_). A log ratio higher than zero indicates male bias. In panels A and B, shading refers to the experimental treatment and indicates the level of stress as measured by reduction in mean fitness: clear for CT (no stress), medium shading for HT (intermediate stress) and dark for AT (high stress).

Variance was calculated from mean standardized fitness. It is therefore the variance in relative fitness, which represents the opportunity for selection. Variance in relative fitness was greater in males than in females in all three treatments (Figs. 4A and 4B). The male bias was largest in the HT, whereas the CT and AT did not differ from each other (HT‐CT: *P* = 0.039; HT‐AT: *P* = 0.039; AT‐CT = 0.45; *P*‐values were obtained from Bayesian posterior distributions, correction for multiple testing using the Bonferroni method).

## Discussion

In sexually reproducing species, selection is often measured to be stronger on males that on females, and this sex bias has often been ascribed to sexual selection acting more on males (Janicke et al. 2016; Singh and Punzalan 2018). This general pattern of stronger selection in males can play an important role in evolution by shaping sexually reproducing populations in many ways, from genetic architecture to mutation load and speed of adaptation. Yet, it is not clear how robust this pattern is to variation in ecological conditions; because the sexes are limited by different resources, variation in sex‐specific limiting resources should alter the sex bias in selection. Using individual‐based simulations of a general model of reproduction, we predicted that imposing female‐specific stress should result in more female‐biased opportunity for selection. Here, we used the model species *C. maculatus* to test this prediction. However, after challenging females by limiting high‐quality oviposition sites (HT) or by delaying age at oviposition (AT), we found that opportunity for selection remained stronger in males and in one case (HT) was even more male biased than in the CT. This result suggests that the trend of stronger opportunity for selection in males is robust to variation at least regarding the environmental variables studied here. One possible explanation that we discuss below is that selection on males is partly mediated by female choice and therefore reflects selection acting on females as well. Additionally, our predictions from individual‐based simulations rely on the assumption that variance in condition is magnified by stress, which may not always be the case. We also discuss the implications of this possibility below.

The two experimental treatments, HT and AT, were designed to be challenging and this is confirmed by our results that show how these stressors decrease mean fitness (adult offspring count) compared to the CT. We also note that on average fitness measured from male assays was higher than from female assays, which may be due to a sex specificity in sensitivity to the irradiation treatment of sterile competitors (we note that male and female assays are symmetric but distinct assays, with consequently no obligation for average male and female fitness to be exactly equal). In any case, this difference is not expected to affect our mean standardized estimates of variance in fitness. A general expectation is that variance in fitness should increase under stressful conditions such as our experimental treatments imposed, as the population is pushed away from its fitness peak (Parsons 1987) and differences among individuals are revealed or magnified (Agrawal and Whitlock 2010). We based our simulations and our predictions on this expectation. However, as outlined by Hoffmann and Merilä (1999), there are scenarios such as severe resource limitation that prevents individuals from expressing their full potential, which allows for a reduction instead of an increase in the opportunity for selection under stress, a prediction that has found some empirical support (reviewed by Agrawal and Whitlock 2010). This is what we also find here: both male and female opportunity for selection decreased under the HT compared to CT, and female variance decreased proportionally more than male variance resulting in a more male‐biased opportunity for selection in that treatment. It is possible that limiting good‐quality larval environment in the HT prevented individuals from achieving their full reproductive potential, thereby decreasing variance in relative fitness at the population level, as predicted by Hoffmann and Merilä (1999). However, if environmental conditions had imposed a ceiling on reproductive performance, we would have expected to see this reflected in the fitness distributions that should have been more negatively skewed in the HT treatment. We did not observe this (skewness score: CT = –0.38, HT = –0.09, AT = 0.17). In fact, the HT treatment of heterogeneous bean quality should not represent an unsurmountable challenge for female *C. maculatus*, as they are known to be capable of complex oviposition decisions (e.g., Mitchell 1975).

Alternatively, it is also plausible that although the HT provided poorer resources that challenged female oviposition strategy and ultimately lowered mean fitness, it may also have removed some of the constraints presented to females in the CT. *Callosobruchus maculatus* is known for pervasive interlocus sexual conflict, where male mating behavior can substantially lower female lifespan and reproductive success (Eady et al. 2006); it is possible that the beans filled with cavities (constituting the majority of the substrate in the HT) offered more hiding opportunities than fresh beans for females to avoid male mating attempts, as adults easily fit in the bean holes made by previous generations (pers. obs.). There is previous evidence suggesting that more complex laboratory environments could reduce the impact of sexual conflict in *Drosophila melanogaster* (Singh et al. 2017). If that is the case here, the HT may have presented females with oviposition challenges but removed or alleviated selection pressure from interlocus sexual conflict. In turn, if the HT made it more difficult for males to find mating partners, this could also explain the stronger male bias in opportunity for selection in that treatment.

In the AT, the opportunity for selection on females increased, as we expected when imposing a challenge on female oviposition strategy (here, age‐at‐reproduction). However, it also increased proportionally in males, which resulted in a sex bias similar to the one measured in the CT. The fact that male opportunity for selection should be affected by female‐limited stress can be expected because male reproductive success remains highly dependent on female fecundity. We see this effect clearly in our individual‐based simulations, where we show that female‐limited stress results in an increase of male opportunity for selection as well, although to a lesser extent than for female opportunity for selection (Fig. 1A). In the simulations, female‐limited stress results in more female‐biased opportunity for selection (Fig. 1B), but what we instead observed here was a proportional increase of opportunity for selection in both males and females, maintaining the sex ratio in opportunity for selection of the CT. We consider several alternative explanations for this result.

Males and females were interacting throughout their lifetime in all of the three treatments; however, in the AT, the oviposition was only possible after 48 h imposing a constraint particularly to the female reproduction. This constraint may have come through ageing, delay between fertilization and oviposition, and increased number of matings before oviposition. In a related seed beetle species (*Acanthoscelides obtectus*), experimental work has shown how selection for a delayed oviposition has resulted in sex‐specific evolution of a number of life history traits, including a female‐biased increase in lifespan (Tucić et al. 1996). However, a constraint to female egg laying is clearly an important factor for males too: there is evidence in *C. maculatus* for last male sperm precedence (Eady 1991), which could have favored males in better condition after 48 h. This environment could have therefore presented an ageing challenge to both sexes. However, even in that case the different reproductive functions are under selection in the sexes, and the effects of ageing are still expected to be sex specific with females being more sensitive than males (Maklakov and Arnqvist 2009; Immonen et al. 2016).

Another possibility is that the challenge imposed on females by the AT was transferred to males through mate choice. This may have happened through several mechanisms related to delayed oviposition, for example, through intense male‐male competition during the period preceding oviposition, or simply with more stringent mate choice of females confronted with a stressful environment. The impact of female condition on mate choice has been studied in many systems; however, the observations mainly support weaker mate choice by females in poor conditions (reviewed by Cotton et al. 2006, and supported by more recent empirical studies Atwell and Wagner Jr 2014; Davis and Leary 2015). Similarly, in the *A. obtectus* seed beetles mate choice becomes relaxed in females when tested in stressful conditions (Stojković et al. 2014). These studies indicate that female‐specific stress reduces rather than increases the strength of selection imposed on males by female choice. However, a different response could be expected if males can contribute to improve female condition through direct benefits such as nuptial gifts or parental care. In *C. maculatus*, male ejaculate represents a large amount of water, carbohydrates, proteins, and peptides, and is sometimes considered a nuptial gift (Edvardsson 2007; Ursprung et al. 2009) in this aphagous species. It is possible that ageing females would rely more on nutrition and hydration from the contributions of male ejaculate to sustain their reproductive capacity. By imposing selection on delayed reproductive ageing, the AT could have resulted in more stringent mate choice imposed on males that could in turn explain the proportional increase of both the male and female variance in fitness. This mechanism could help to explain the maintenance of stronger opportunity for selection in males even under the limitation of female‐specific resources at least in species where mating provides direct resource benefits to females.

## Conclusions

We have shown that there are sex‐specific changes in the opportunity for selection in response to different ecological challenges. Although this has been tested before (e.g., Janicke et al. 2015; Martinossi‐Allibert et al. 2017), in the present study we placed particular focus on female‐specific resource limitation, with the prediction that it would lead to a more female‐biased opportunity for selection. This prediction relied on the assumption that resource limitation would generally increase opportunity for selection. Despite the variety of ways in which sex‐specific selection responded to our different treatments, opportunity for selection remained stronger in males in all cases, which suggests that this pattern is in fact relatively robust. Moreover, our results from the HT showed that a male bias in the opportunity for selection can also be driven by a response of females to changes in environmental conditions, which challenges the view that stronger opportunity for selection in males is generally driven by intense sexual competition in males. Although it is not surprising that manipulating variance in fitness of one sex should trigger a response in the other because of the many levels of interactions involved in sexual reproduction, it is rather striking that males remained the more variable sex regardless of the degree of stress on females.

## AUTHOR CONTRIBUTIONS

EI, JR, and IM conceived and designed the project. IM and JR conducted the experiments. IM analyzed the data and wrote the manuscript with help from EI.

## DATA ARCHIVING

The data supporting this research can be found at https://doi.org/10.5061/dryad.08kprr510.

Associate Editor: S. Glemin

Handling Editor: D. W. Hall

## Supporting information


**Figure 1**. The population is represented by two independent normal distributions of conditions for females and males, with mean 1 and variance respectively *V*(*c*_f_) and *V*(*c*_m_).
**Figure 2**. Proportion of resources obtained by the focal individual as a function of its condition and of the competition parameter *g*, considering a competitor of condition 1 (population average).
**Figure 3**. Proportion of female eggs fertilized as a function of male mean condition and the male contribution to fertility parameter *mc*.
**Figure 4**. Opportunity for selection in males *(I*_m_ *, circles)* and females *(I*_f_ *, diamonds)* with increasing stress for both sexes (a), females only (b) and males only (c) for the male‐biased competition scenario with nuptial gifts.
**Figure 5**. Log‐ratio of the opportunity for selection in males over females *(I*_m_ */I*_f_ ) with increasing stress for both sexes (black), females only (purple) and males only (brown) for the male‐biased competition scenario with nuptial gifts.
**Figure 6**. Opportunity for selection in males *(I*_m_ *, circles)* and females *(I*_f_ *, diamonds)* with increasing stress for both sexes (a), females only (b) and males only (c) for the male‐biased competition scenario with male harm.
**Figure 7**. Log‐ratio of the opportunity for selection in males over females *(I*_m_ */I*_f_ ) with increasing stress for both sexes (black), females only (purple) and males only (brown) for the male‐biased competition scenario with male harm.
**Figure 8**. Opportunity for selection in males *(I*_m_ *, circles)* and females *(I*_f_ *, diamonds)* with increasing stress for both sexes (a), females only (b) and males only (c) for the male‐biased competition scenario with sperm limitation.
**Figure 9**. Log‐ratio of the opportunity for selection in males over females *(I*_m_ */I*_f_ ) with increasing stress for both sexes (black), females only (purple) and males only (brown) for the male‐biased competition scenario with sperm limitation.
**Figure 10**. Opportunity for selection in males *(I*_m_ *, circles)* and females *(I*_f_ *, diamonds)* with increasing stress for both sexes (a), females only (b) and males only (c) for the scenario with equal competition across sexes.
**Figure 11**. Log‐ratio of the opportunity for selection in males over females *(I*_m_ */I*_f_) with increasing stress for both sexes (black), females only (purple) and males only (brown) for the scenario with equal competition across sexes.

Supplementary Material

Supplementary Material
