# Supplementary material for: Female‐specific resource limitation does not make the opportunity for selection more female biased
Source: Evolution. 2020 Oct 20;74(12):2714–24. doi: 10.1111/evo.14106 (PMC7821317; doi:10.1111/evo.14106)
Supplement: Supplementary file 1 — Figure 1. The population is represented by two independent normal distributions of conditions for females and males, with mean 1 and variance respectively V(c f) and V(c m). Figure 2. Proportion of resources obtained by the focal individual as a function of its condition and of the competition parameter g, considering a competitor of condition 1 (population average). Figure 3. Proportion of female eggs fertilized as a function of male mean condition and the male contribution to fertility parameter mc. Figure 4. Opportunity for selection in males (I m , circles) and females (I f , diamonds) with increasing stress for both sexes (a), females only (b) and males only (c) for the male‐biased competition scenario with nuptial gifts. Figure 5. Log‐ratio of the opportunity for selection in males over females (I m /I f ) with increasing stress for both sexes (black), females only (purple) and males only (brown) for the male‐biased competition scenario with nuptial gifts. Figure 6. Opportunity for selection in males (I m , circles) and females (I f , diamonds) with increasing stress for both sexes (a), females only (b) and males only (c) for the male‐biased competition scenario with male harm. Figure 7. Log‐ratio of the opportunity for selection in males over females (I m /I f ) with increasing stress for both sexes (black), females only (purple) and males only (brown) for the male‐biased competition scenario with male harm. Figure 8. Opportunity for selection in males (I m , circles) and females (I f , diamonds) with increasing stress for both sexes (a), females only (b) and males only (c) for the male‐biased competition scenario with sperm limitation. Figure 9. Log‐ratio of the opportunity for selection in males over females (I m /I f ) with increasing stress for both sexes (black), females only (purple) and males only (brown) for the male‐biased competition scenario with sperm limitation. Figure 10. Opportunity for selection in males (I m , circles) and females (I f , diam [file EVO-74-2714-s001.docx]

**Supplementary file S1:**

**Individual-based simulation of sex-specific opportunity for selection.**

Contents

[Aims of the simulations and main conclusion: 2](#_Toc50129496)

[Features of the model: 2](#_Toc50129497)

[Individual condition 2](#_Toc50129498)

[Simulation of sex-specific stress 3](#_Toc50129499)

[Sexual reproduction 3](#_Toc50129500)

[Female fitness with female competition, nuptial gifts, male harm and sperm limitation 3](#_Toc50129501)

[Male fitness as a function of male condition and female fecundity 5](#_Toc50129502)

[Running the individual based simulations 6](#_Toc50129503)

[Results 6](#_Toc50129504)

[Male-biased competition (no harm, no nuptial gifts, no sperm limitation) 6](#_Toc50129505)

[Male-biased competition with nuptial gifts 6](#_Toc50129506)

[Male-biased competition with male harm during mating (sexual conflict) 8](#_Toc50129507)

[Male-biased competition with sperm limitation 9](#_Toc50129508)

[Competition in both sexes (no harm, no nuptial gifts, no sperm limitation) 11](#_Toc50129509)

[Conclusion 12](#_Toc50129510)

[References 13](#_Toc50129511)

# Aims of the simulations and main conclusion:

In the main manuscript, we lay out the core of the model that we used to simulate sex-specific opportunity for selection under stress. In this baseline model, we made the assumptions that males only compete for mating, and that no beneficial or harmful interactions between males and females occur during mating, nor does sperm limitation. Here in this supplementary file, we relax these assumptions and examine how these dynamics affect the predictions.

The baseline scenario, presented in the main manuscript, predicted that sex-limited stress should bias the opportunity for selection towards the more stressed sex, and that stressing both sexes equally resulted in a *status quo*. Here, we examine how this key result is affected by additional aspects of reproduction: female-female competition and female-male interactions (nuptial gifts, male harm and sperm limitation). We find that nuptial gifts only matter in the case of male-limited stress, resulting in a less male-biased opportunity for selection than in the case without nuptial gifts. If female fitness is more dependent on male condition, then it seems intuitive that male –specific stress should affect females as well. The effect of sperm limitation is somewhat similar to the effect of nuptial gifts, which makes sense because they both entail a positive contribution of male condition to female fitness. If male harm occurs during mating, meaning that female fecundity is reduced proportional to average male condition, then opportunity for selection becomes closer to equal across the sexes, regardless of onto which sex stress is applied. This occurs because selection becomes stronger on both sexes, reducing the relative difference between the sexes.

# Features of the model:

Here, we come back to the features of the model already described in the main manuscript, with additional details and figures, and we also expand to include the additional features of female-female competition, sexual interactions (male harm or nuptial gifts) and sperm limitation.

The process of sexual reproduction takes into account condition-dependent female fecundity, and condition-dependent male and female intrasexual competition. We also consider the possibility for sperm limitation, male harm during mating and nuptial gifts.

Individuals are described by a single variable, relative condition, which determines both their fecundity or fertility and competitive ability. We consider this to be a reasonable assumption for a model that is primarily aimed at studying sexual selection, where comparing individuals in terms of relative quality is the main concern. This is because traits determining reproductive fitness are typically condition dependent, and therefore highly polygenic, which leads to the expectation that phenotypic fitness variation subject to condition reflects overall genetic variation in individual quality (Rowe and Houle 1996).

## Individual condition

The condition of each individual is drawn from a normal distribution of mean 1. The mean is always fixed to one, because the condition is relative within each sex. The standard deviation of the normal distribution is variable according to the level of stress (see Figure 1).

## Simulation of sex-specific stress

Stress is simulated by increasing the standard deviation of the distribution of conditions. This represents the expression of cryptic genetic variants revealed by a new or stressful environments. The standard deviation of the distribution of conditions can be varied independently for each sex (see Figure 1).


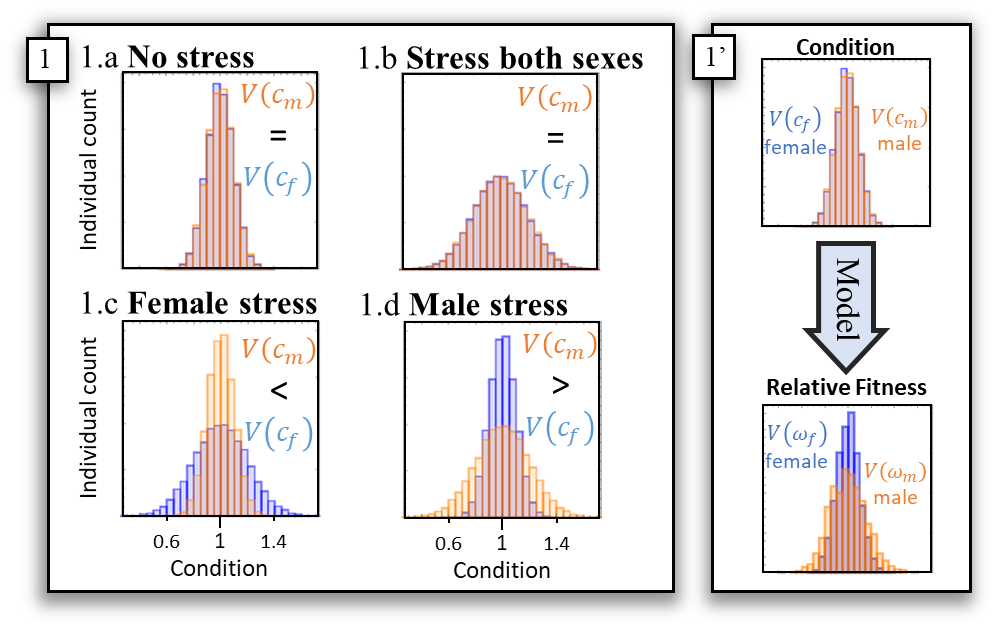


**Figure 1.** The population is represented by two independent normal distributions of conditions for females and males, with mean 1 and variance respectively$V\left( c_{f} \right)$ and $V\left( c_{m} \right)$. Stress is represented by an increase in variance in condition. Stress can vary independantly for each sex. In panel 1’, we show how the sex-specific condition distribution is translated into a sex-specific fitness distribution by the model of sexual reproduction (case with male-male competition).

## Sexual reproduction

To mimic closely the experimental design that the present simulation makes prediction for, we consider sexual reproduction in groups of four individuals, two of each sex. The two females have condition ${cf}_{1}$ and ${cf}_{2}$. The two males have condition ${cm}_{1}$ and ${cm}_{2}$.

## Female fitness with female competition, nuptial gifts, male harm and sperm limitation

Each female is able to produce a number of fertile eggs which depends on: her condition ${cf}_{1}$, the outcome of female-female competition, and potentially harmful or beneficial interactions with males during mating. We assume that female fecundity scales linearly with female condition. Females can engage in competition for resources, which we assume to be condition dependent. The intensity of this competition follows the parameter $gf$, with $gf=0$ indicating no competition. The proportion of resources secured by the focal female of condition ${cf}_{1}$ is defined by the competition function (see Figure 2):

$$\frac{{{cf}_{1}}^{gf}}{{{cf}_{1}}^{gf}+{{cf}_{2}}^{gf}}.$$

The contribution of males to female fecundity is dependent on the parameter $imf$ (for interaction male-female). $imf$ is comprised between -1 and 1 and indicates if interactions are positive ( $0<imf\leq1$: nuptial gift or parental care, increasing female fecundity) or negative ( $-1\leq imf<0$: interlocus sexual conflict, male harm during mating). $imf=0$ indicates that males do not affect female fecundity. The effect of males on female fecundity is also weighted by average male condition within the group. Taking all of this into account, female fecundity, or the number of fertile eggs that a female can produce is:

$$\mathrm{Eggs}={cf}_{1}\times\frac{{{cf}_{1}}^{gf}}{{{cf}_{1}}^{gf}+{{cf}_{2}}^{gf}}\times\left( 1+imf\times\frac{({cm}_{1}+{cm}_{2})}{2} \right)$$

Finally, the fitness of the focal female is the proportion of these fertile eggs that gets fertilized by males, according to average male condition and the parameter $mc$, (for male contribution to fertility).

$$Female fitness=\mathrm{Eggs}\times\frac{\frac{{cm}_{1}+{cm}_{2}}{2}}{mc+(1-mc)\frac{{cm}_{1}+{cm}_{2}}{2}}$$

If we consider that sperm is not limiting ($mc=0$), then the second term becomes 1 and all eggs are fertilized automatically regardless of the condition of the males. If $0<mc<1$ however, female fitness becomes an increasing and saturating function of average male condition. If $mc=1$, then female fitness scales linearly with average male condition. The behaviour of the female competition function and the male contribution to fertility can be found in Figures 2 and 3.


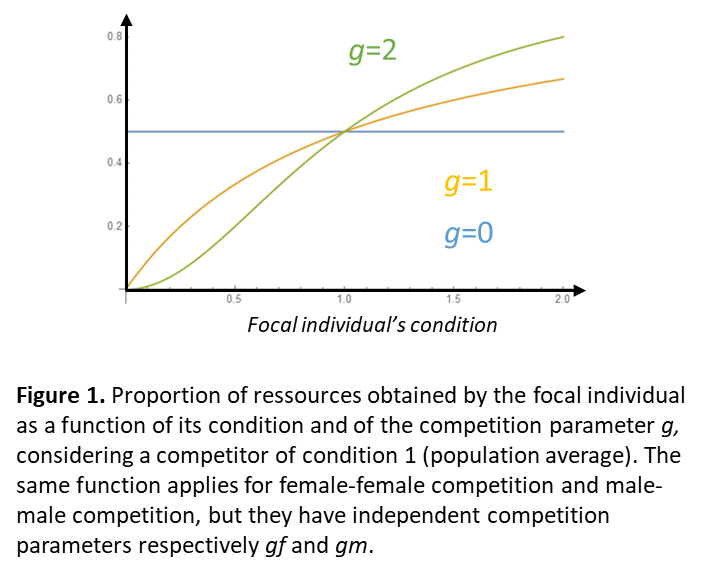


**Figure 2.** Proportion of resources obtained by the focal individual as a function of its condition and of the competition parameter *g,* considering a competitor of condition 1 (population average). The same function applies for female-female competition and male-male competition, but they have independent competition parameters respectively *gf* and *gm*.


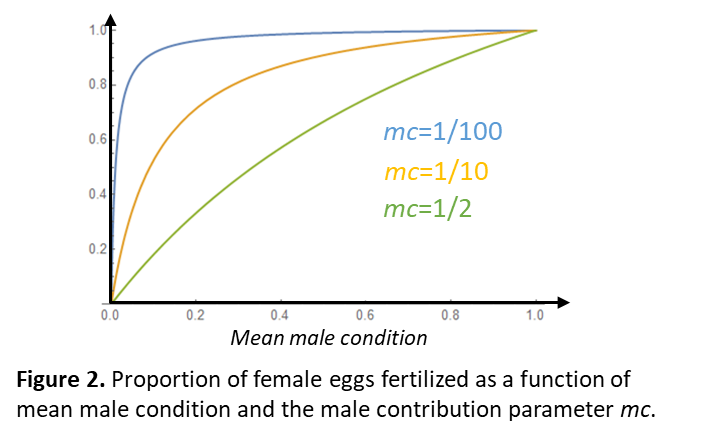


**Figure 3.** Proportion of female eggs fertilized as a function of male mean condition and the male contribution to fertility parameter *mc*.

## Male fitness as a function of male condition and female fecundity

In each group of four individuals, the two males share the total fecundity of the two females, as calculated by the female fitness function presented above. Males compete for their portion of fecundity, using the same condition dependent functions that females use to compete (see Figure 2). Note however that the intensity of this competition is independent across the sexes, so that it is possible to parametrize the model for one sex only to compete.

$$Male fitness=Total female fitness\times\frac{{{cm}_{1}}^{gm}}{{{cm}_{1}}^{gm}+{{cm}_{2}}^{gm}}$$

# Running the individual based simulations

We use the software Wolfram Mathematica to sample individual condition and implement the sexual reproduction model, as well as for production of graphical output. The Mathematica code used to produce the results of this appendix as well as the Figures of Box 1 in the main manuscript can be found in Supplementary file S2.

We investigate several scenarios of sexual reproduction and show how they affect the sensitivity of sex-specific opportunity for selection to stress, as defined here by an increase in the variance in condition. For each scenario, we show how opportunity for selection for each sex independently reacts to (i) stress on both sexes, (ii) stress on females only, and (iii) stress on males only, and finally how each of these stress gradients affects the sex-ratio of opportunity for selection.

For each simulation run, a population size of 1000 is assumed. Each sex-specific graph (Figures 4,6,8,10) presents the average of 10 simulations per sex and per scenario. Sex-ratio graphs (Figures 5,7,9,11) present the average of 20 repeated simulations per scenario. On each graph, the “Stress” axis gives the value of the standard deviation of the distribution of individual condition in the population, around a fixed mean of 1.

# Results

## Male-biased competition (no harm, no nuptial gifts, no sperm limitation)

See main manuscript.

| **Parameter values** |
| --- |
| *gf=0*  ***gm=2***  *imf=0*  *mc=1/100* |

## Male-biased competition with nuptial gifts

Adding nuptial gifts offers the possibility for male condition to contribute positively to the fecundity (egg production) of females, instead of simply to egg fertilization. A parameter *imf* of value +½ means that the contribution of an average male of condition 1 can raise the fecundity of the mated female by half of its original value which itself depends on the female’s own condition.

| **Parameter values** |
| --- |
| *gf=0*  ***gm=2***  ***imf=1/2***  *mc=1/100* |

We can see on Figure 4 that in this new scenario there is little change in the case of stress applied to box sexes or to females only, as compared with the previous scenario. In the case of male limited stress however, we can see that female opportunity for selection is now more affected by male-limited stress than it was before. This is expected, as female fitness now depends on male condition to some extent. As a consequence of this, under male-limited stress, the sex-ratio of opportunity for selection does not become as male-biased as in the original scenario without nuptial gifts (Figure 5).


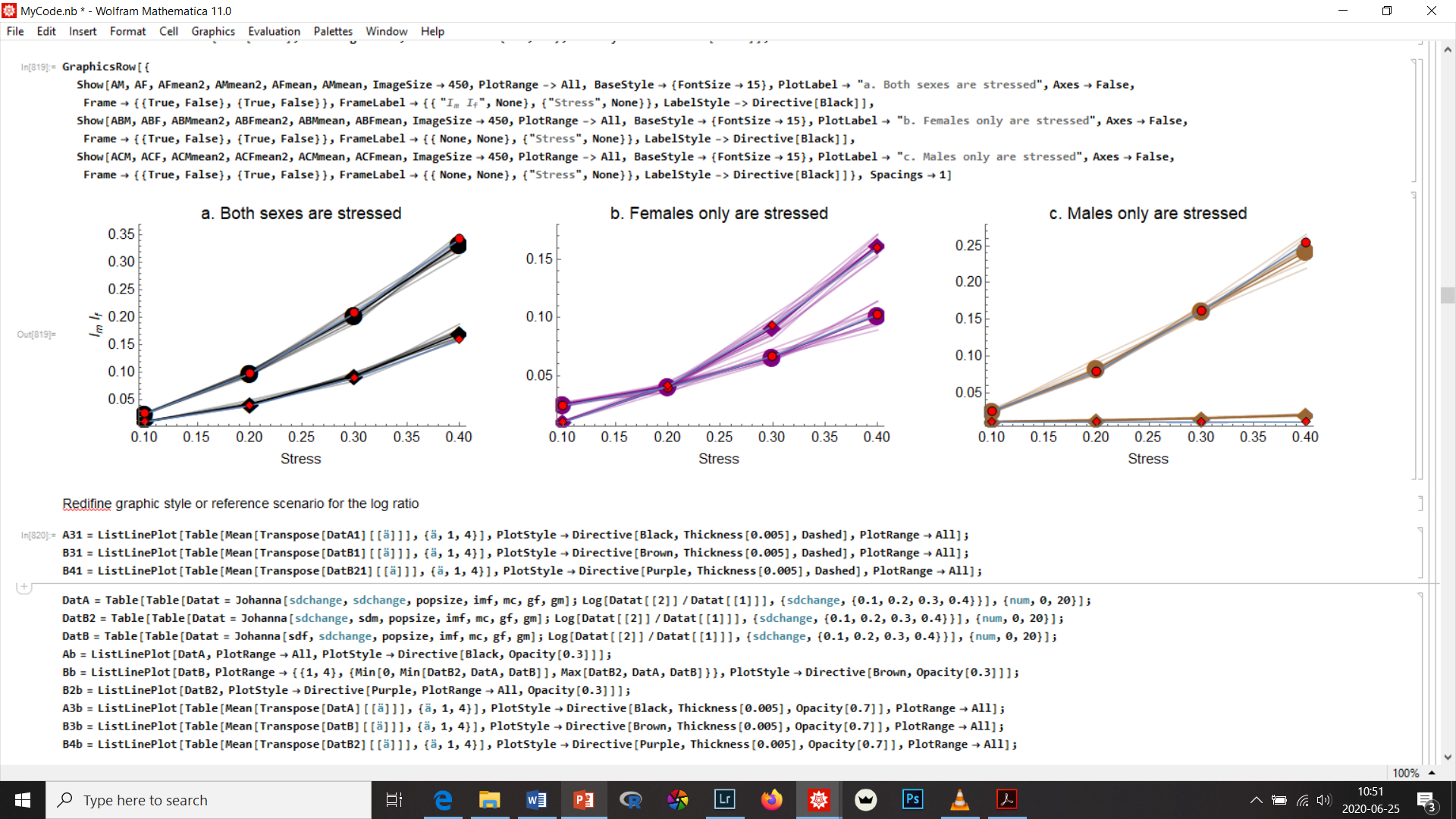


**Figure 4.** Opportunity for selection in males *(I_m_ , circles)* and females *(I_f_ , diamonds)* with increasing stress for both sexes (a), females only (b) and males only (c) **for the male-biased competition scenario with nuptial gifts**. Transparent lines represent individual simulations and thick lines the average of these 10 replicates. Red markers represent the average of the original male biased competition scenario without nuptial gifts for comparison.


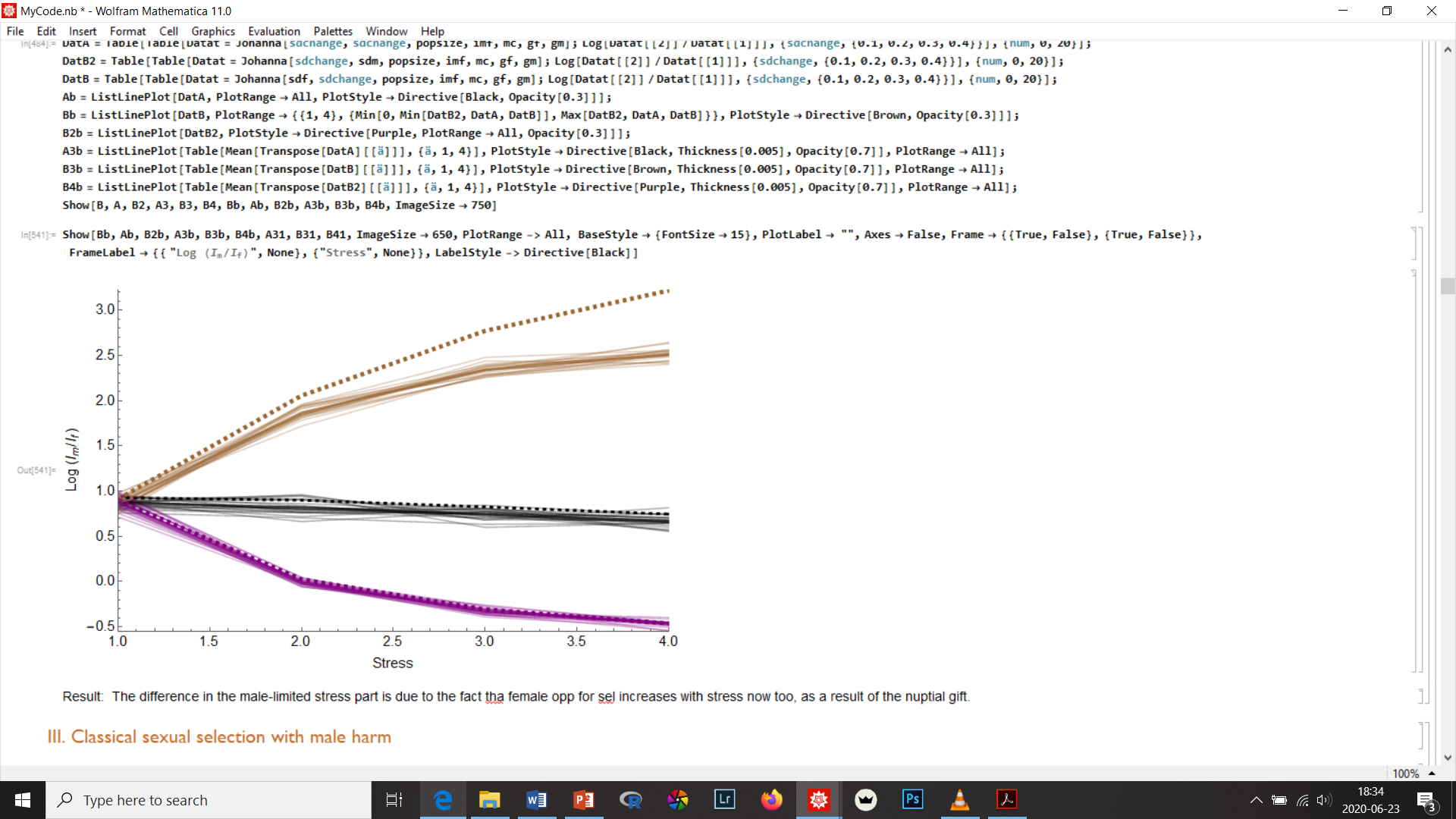


**Figure 5.** Log-ratio of the opportunity for selection in males over females *(I_m_/I_f_* ) with increasing stress for both sexes (black), females only (purple) and males only (brown) **for the male-biased competition scenario with nuptial gifts**. A log-ratio of zero indicates no sex bias. Transparent lines represent individual simulations and thick lines the average of these 20 replicates. Dashed lines represent the average of the original male biased competition scenario without nuptial gifts for comparison.

## Male-biased competition with male harm during mating (sexual conflict)

Adding male harm means that female fecundity (egg production) is negatively affected by male condition. With a parameter *imf* of value -½, this means that an average male of condition 1 can half female egg production. Arguably, the intensity of male harm may be stronger in this scenario than what can be found in nature, but the aim here is to provide general qualitative predictions on the effect of male harm on sex-specific opportunity for selection.

| **Parameter values** |
| --- |
| *gf=0*  ***gm=2***  ***imf= - 1/2***  *mc=1/100* |

On Figure 6, we can see that a general effect of male harm is to increase selection for both sexes, with the weakest effect observed for female-limited stress. To put it simply, stress on males, which is modelled here by an increase in variance in condition, results in an increase in the opportunity for selection in males both through direct effects on male fitness and indirect effects through female fecundity. Females are also affected because male harm implies an impact of male condition on female fitness. An important caveat to point out here is that if we had chosen to model stress differently, for example by a decrease in mean condition, we would probably have observed a reduction of male harm with male-specific stress and therefore weaker instead of stronger selection on females in that case.


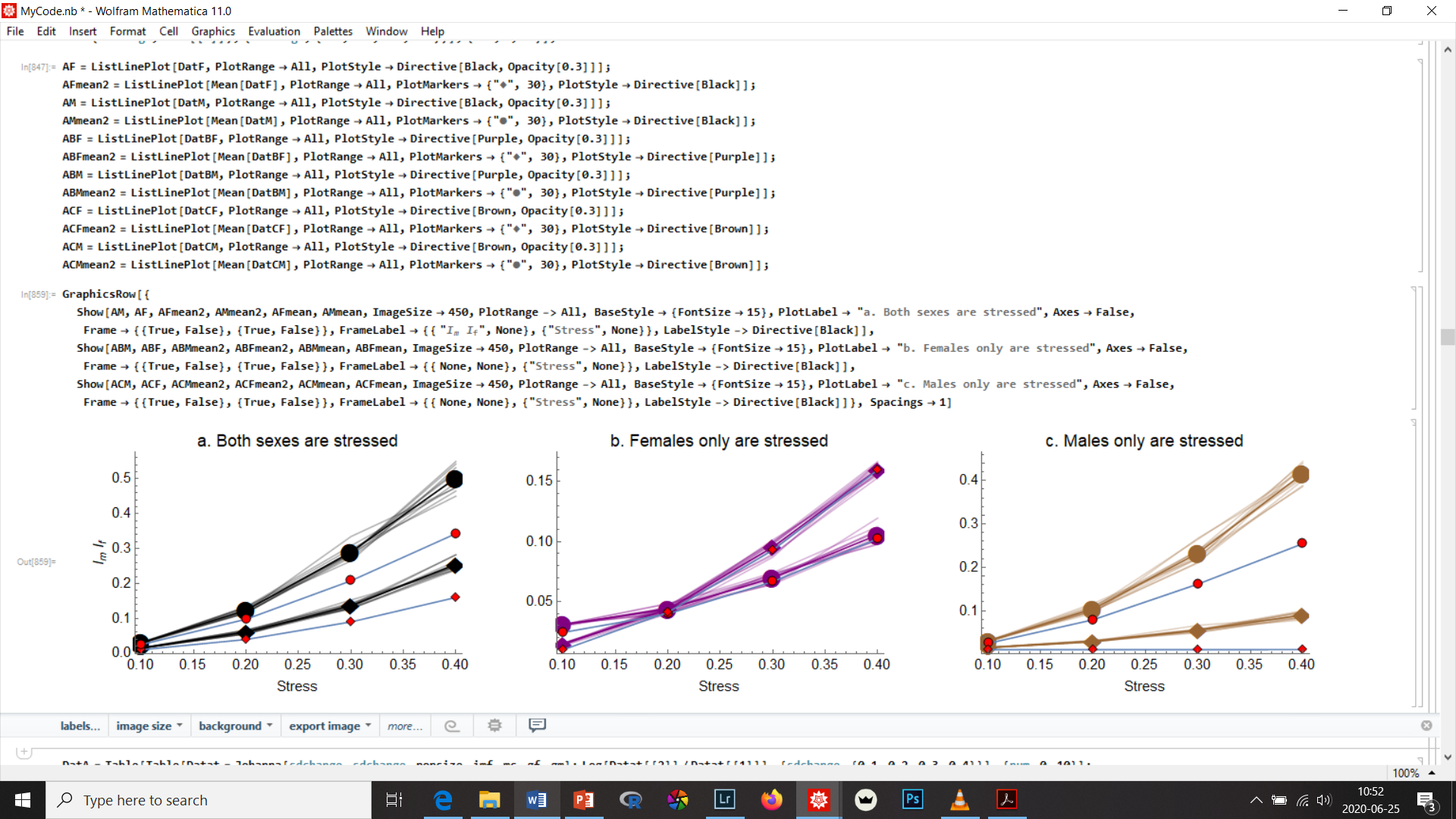


**Figure 6.** Opportunity for selection in males *(I_m_ , circles)* and females *(I_f_ , diamonds)* with increasing stress for both sexes (a), females only (b) and males only (c) **for the male-biased competition scenario with male harm**. Transparent lines represent individual simulations and thick lines the average of these 10 replicates. Red markers represent the average of the original male biased competition scenario without male harm for comparison.

Figure 7 confirms that male harm tends to make the opportunity for selection less male-biased.


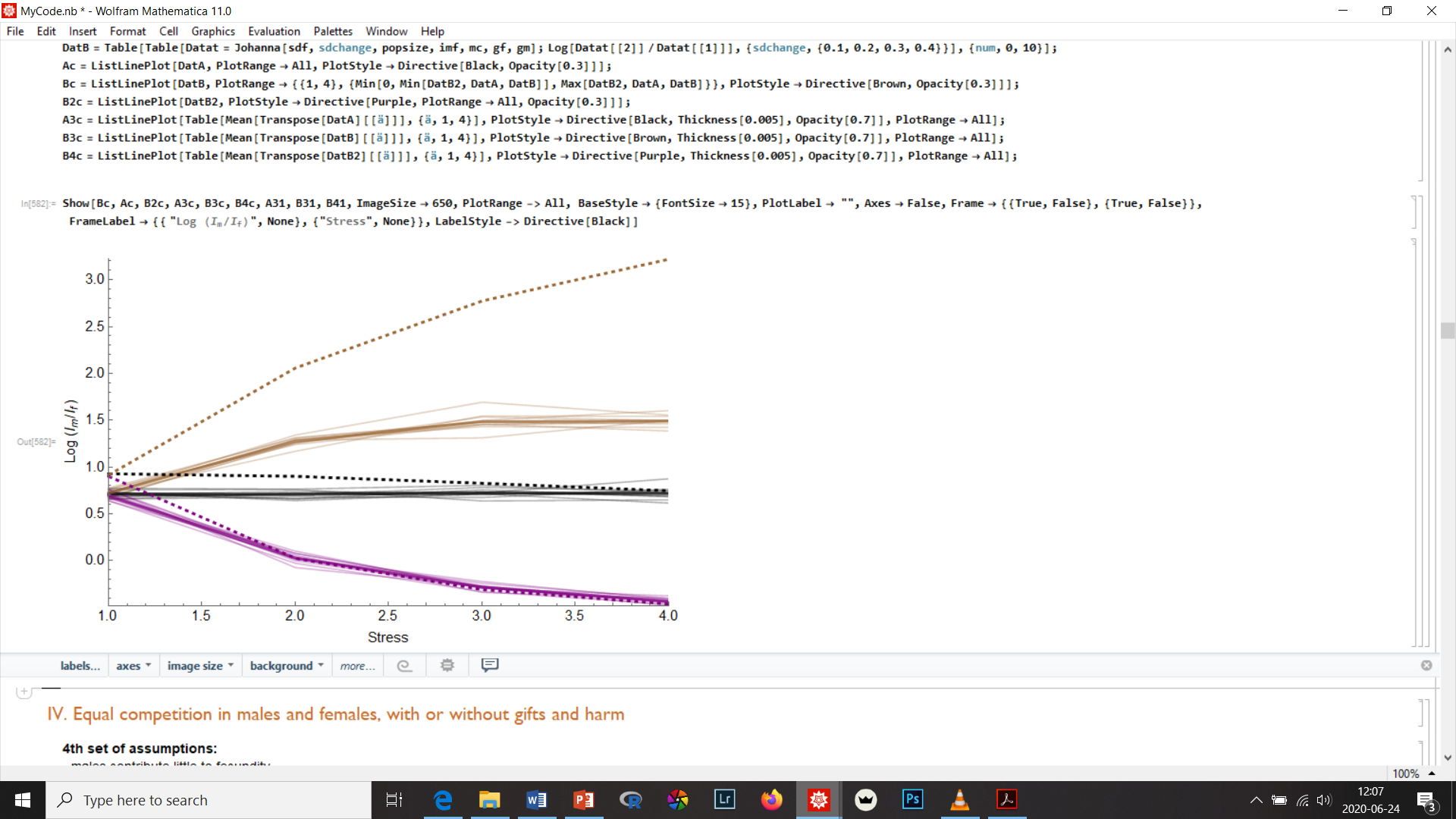


**Figure 7.** Log-ratio of the opportunity for selection in males over females *(I_m_/I_f_* ) with increasing stress for both sexes (black), females only (purple) and males only (brown) **for the male-biased competition scenario with male harm**. A log-ratio of zero indicates no sex bias. Transparent lines represent individual simulations and thick lines the average of these 20 replicates. Dashed lines represent the average of the original male biased competition scenario without male harm for comparison.

## Male-biased competition with sperm limitation

With sperm limitation, male condition does not affect female egg production but it can limit female fitness through fertilisation success. In consequence, we can expect an effect qualitatively similar to nuptial gifts.

| **Parameter values** |
| --- |
| *gf=0*  ***gm=2***  *imf= 0*  ***mc=1/5*** |

Indeed, comparing Figures 8 and 9 with Figures 4 and 5, we can see a similar effect to the nuptial gifts, with the male-bias of opportunity for selection being reduced when males only are stressed as compared to the original scenario without sperm limitation. Again, this occurs because female fitness is now affected as well by male condition, whereas it was not in the original scenario, which made females insensitive to male-limited stress.


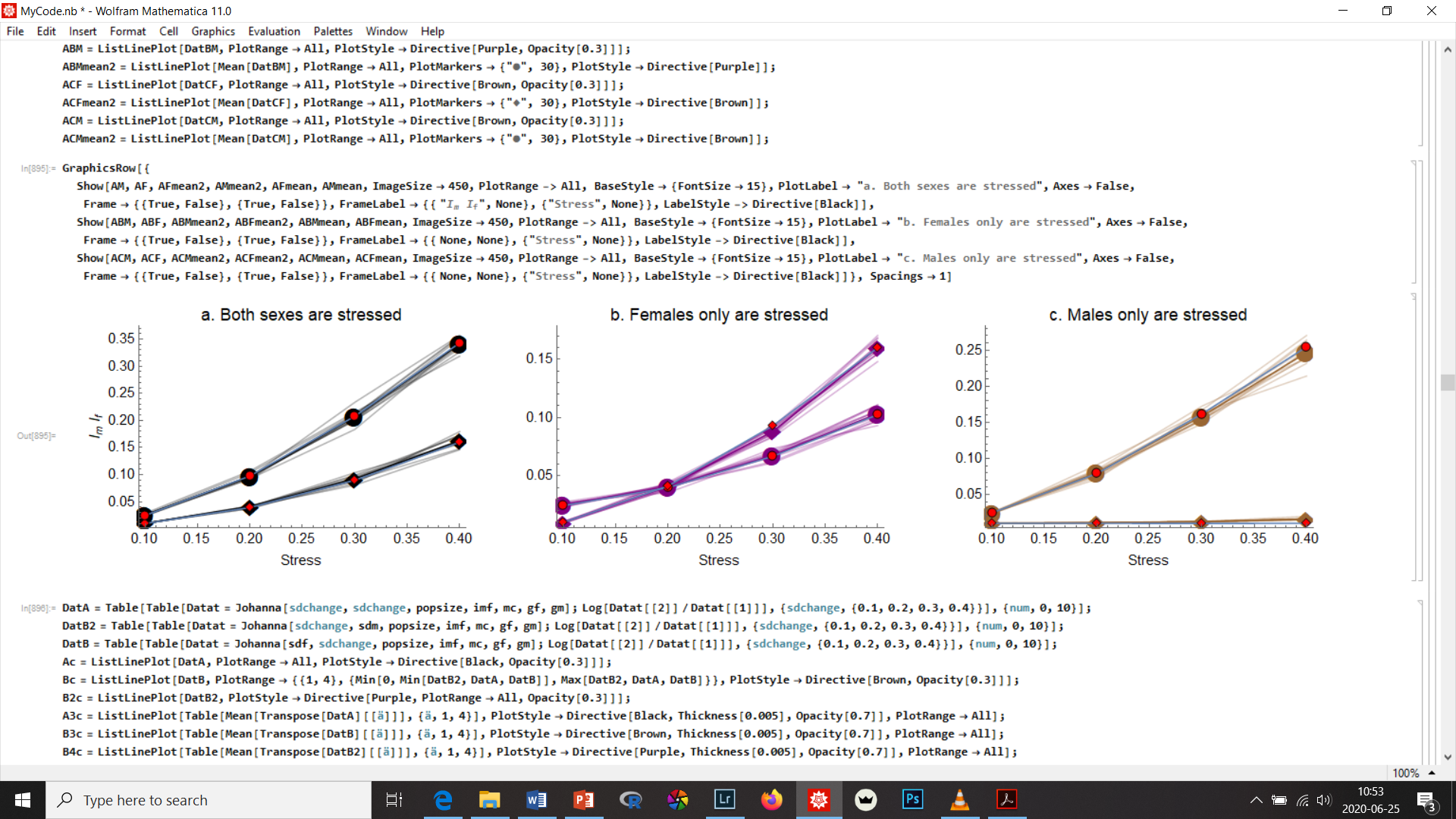


**Figure 8.** Opportunity for selection in males *(I_m_ , circles)* and females *(I_f_ , diamonds)* with increasing stress for both sexes (a), females only (b) and males only (c) **for the male-biased competition scenario with sperm limitation**. Transparent lines represent individual simulations and thick lines the average of these 10 replicates. Red markers represent the average of the original male biased competition scenario without sperm limitation for comparison.


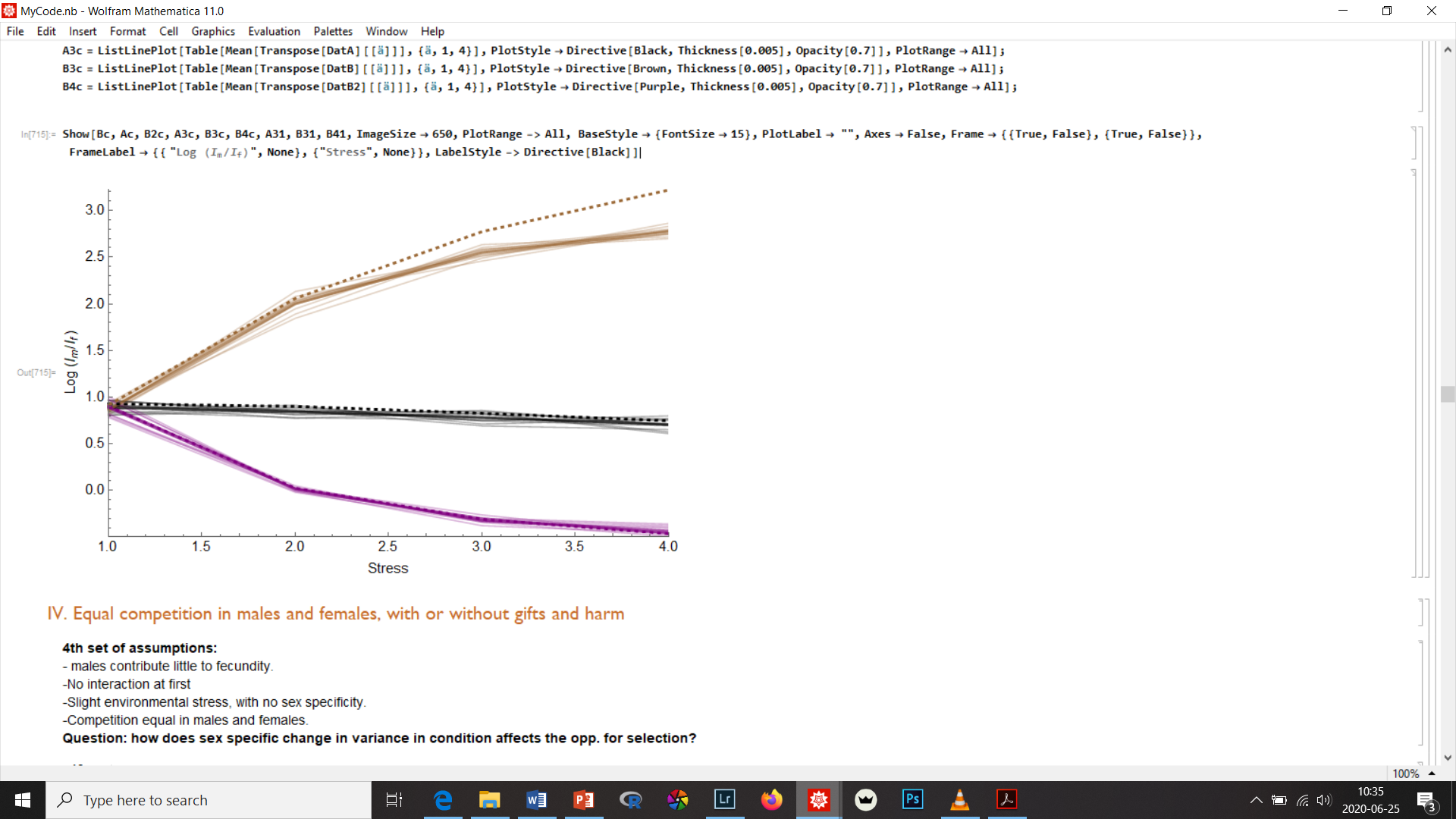


**Figure 9.** Log-ratio of the opportunity for selection in males over females *(I_m_/I_f_* ) with increasing stress for both sexes (black), females only (purple) and males only (brown) **for the male-biased competition scenario with sperm limitation**. A log-ratio of zero indicates no sex bias. Transparent lines represent individual simulations and thick lines the average of these 20 replicates. Dashed lines represent the average of the original male biased competition scenario without sperm limitation for comparison.

## Competition in both sexes (no harm, no nuptial gifts, no sperm limitation)

In this scenario, we allow females to compete with each other to the same extent that males do with each other. In nature, this competition may arise from limitation of resources or nesting or oviposition sites, as well as from competition for mating partners. We do not introduce nuptial gifts or male harm, or sperm limitation in this scenario.

| **Parameter values** |
| --- |
| ***gf=2***  ***gm=2***  *imf= 0*  *mc=1/100* |

It is interesting to notice that although the competition function is the same in males and females, the asymmetry of sexual reproduction results in the opportunity for selection being stronger in females than in males when the competition intensity parameter is the same across sexes. This is easily visualized on Figure 11, where the opportunity for selection is clearly female-biased in the absence of stress.


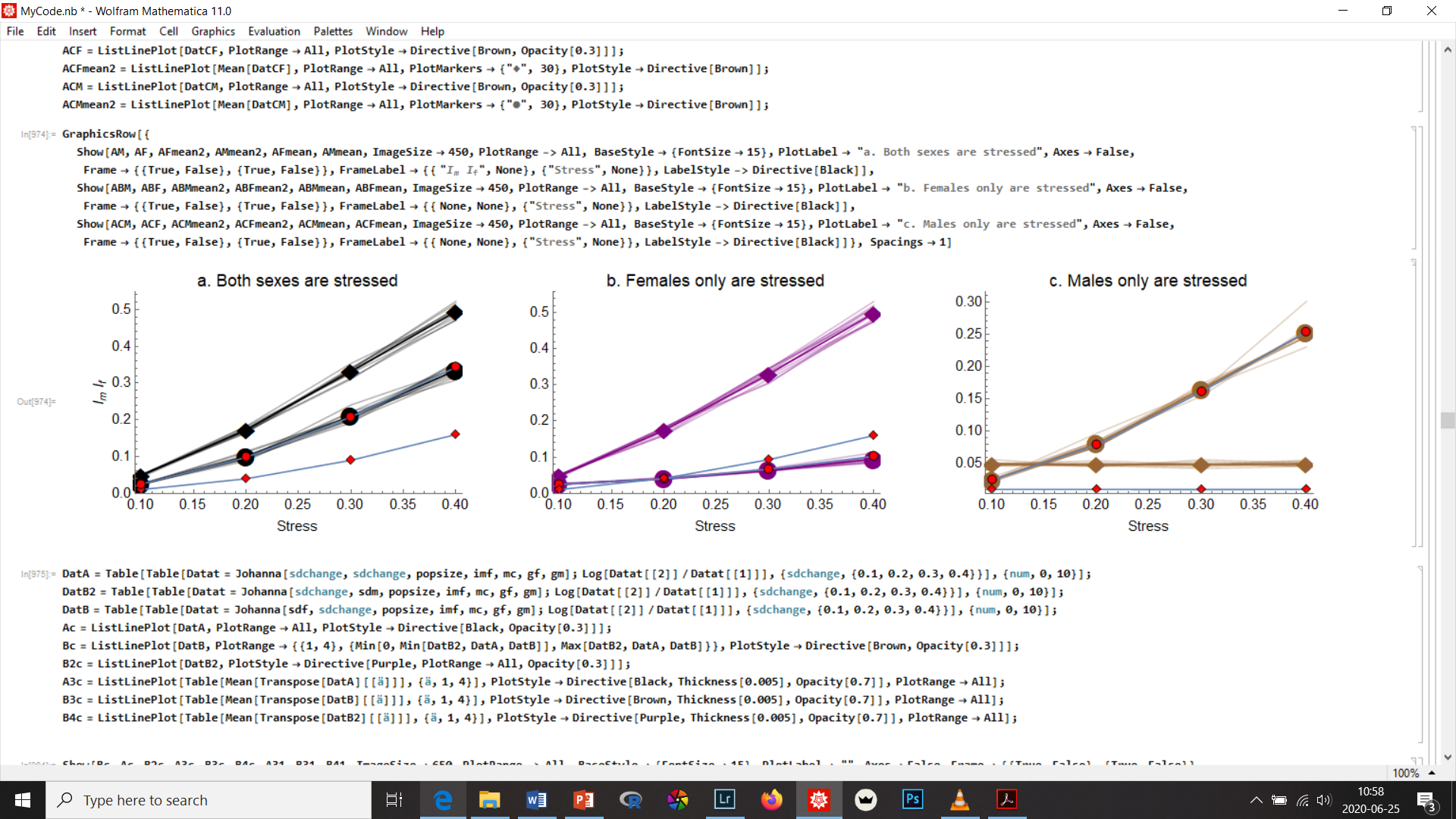


**Figure 10.** Opportunity for selection in males *(I_m_, circles)* and females *(I_f_ , diamonds)* with increasing stress for both sexes (a), females only (b) and males only (c) **for the scenario with equal competition across sexes**. Transparent lines represent individual simulations and thick lines the average of these 10 replicates. Red markers represent the average of the original male biased competition scenario for comparison.

Overall, the response of both sexes to the different types of stress is very similar to the one observed in the original scenario of male-biased competition, with the only notable difference that all the values are shifted towards more female bias (Figure 11). We find again that female-limited stress makes the opportunity for selection more female-biased, and that male-limited stress does not affect selection in females (expected since nuptial gifts, male harm and sperm limitation are absent from this scenario).

89
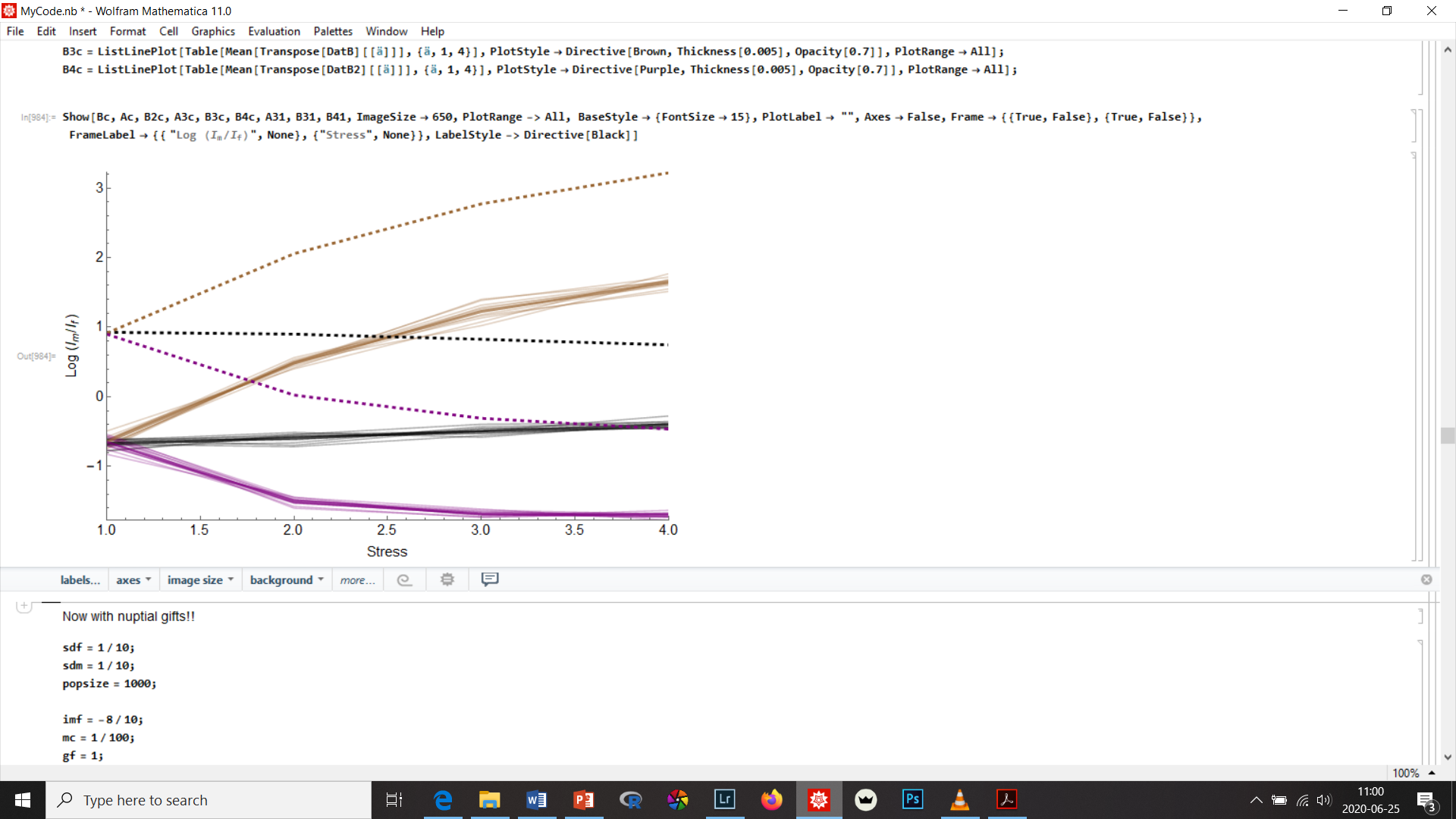


**Figure 11.** Log-ratio of the opportunity for selection in males over females *(I_m_/I_f_* ) with increasing stress for both sexes (black), females only (purple) and males only (brown) **for the scenario with equal competition across sexes**. A log-ratio of zero indicates no sex bias. Transparent lines represent individual simulations and thick lines the average of these 20 replicates. Dashed lines represent the average of the original male biased competition scenario for comparison.

# Conclusion

We use individual based simulations to generate predictions of how environmental stress independently applied to each sex may affect the opportunity for selection in each sex. We find that whenever female-limited stress is applied, the opportunity for selection becomes more female-biased, and this result is consistent over a variety of scenarios including nuptial gifts, male harm, sperm limitation, male-biased competition and equal competition across the sexes. This result is the main finding that we want to highlight and that we use as the baseline prediction for the main manuscript. In addition, we find that nuptial gifts, male harm and sperm limitation make females more sensitive to stress on males, which is expected as female fitness depends on male condition to some extent in those scenarios.

Limitations:

1. The simulation results that we present here aim to generate predictions for the experimental part of the main manuscript, and we have therefore parameterised our models to match our experimental set up. In particular, the fact that reproduction always occurs in groups of four individuals with equal sex-ratio is a characteristic that matches our empirical design well but limits broader generalisations.
2. Our results rely on the assumption that stress can be modelled by an increase in variance in condition without change to the mean. This model may be a good representation of stress when individuals compete within their sex, as the fixed mean condition represents a relative condition within the sex, but it may not be realistic when male harm or nuptial gifts occur if these affect the mean as well.

# References

Rowe, L., & Houle, D. (1996). The lek paradox and the capture of genetic variance by condition dependent traits. *Proceedings of the Royal Society of London. Series B: Biological Sciences*, *263*(1375), 1415-1421.
