# Supplementary material for: Female‐specific resource limitation does not make the opportunity for selection more female biased
Source: Evolution. 2020 Oct 20;74(12):2714–24. doi: 10.1111/evo.14106 (PMC7821317; doi:10.1111/evo.14106)

Supplementary File 2: Mathematica code for individual based simulation of sex-sepcific opportunity for selection.

Creating a population: drawing male and female conditions from a distribution

Mean condition is 1, SD 0.1.

```
malecond = RandomVariate[NormalDistribution[1, 0.1], 100];
femalecond = RandomVariate[NormalDistribution[1, 0.1], 100];
```

```
Mean[malecond]
Mean[femalecond]
```

```
1.00765
```

```
0.995031
```

Fitness functions of females and males

1. Female fitness is a function of female condition. And other stuff.
First, female condition is converted in the number of eggs that the female can produce.
The condition of the focal female, which represents the maximum number of eggs that her genetic makeup should allow her to produce, is multiplied by a competition factor that takes into account the other female's condition. This term represents the possibility for resource competition among females, and its strength can be modulated by parameter gf.
We assume fecundity to scale linearly with condition.
Finally, the number of eggs obtain from the product of condition and competition can be augmented or reduced by a contribution from males (e.g. nuptial gift or male harm), which is dependent on male mean condition and imf, the interaction term that dictates the direction and intensity of male contribution.
condition of female 1: cf1
condition of male 1: cm1
imf: male female interaction term

mc:male contribution to female fecundity.

$$\text{Eggs[cf1\_, cf2\_, cm1\_, cm2\_, imf\_, gf\_] := cf1} * \frac{\text{cf1}^{\text{gf}}}{\text{cf1}^{\text{gf}} + \text{cf2}^{\text{gf}}} * \left(1 + \text{imf} * \frac{(\text{cm1} + \text{cm2})}{2}\right)$$

Female fitness is then the product of the number of eggs defined above and male contribution to fertilization. Male contribution is condition dependent, but can be defined so that male condition does not lower female fertility unless it is extremely low. In other words, this second term defines the intensity of sperm limitation for female fitness. If the parameter mc is low, then male condition matters little for female fitness, but if mc is high then male condition can be as important as female condition.

$$\text{Femfit[cf1\_, cf2\_, cm1\_, cm2\_, imf\_, gf\_, mc\_] :=}$$
$$\text{Eggs[cf1, cf2, cm1, cm2, imf, gf]} * \frac{\frac{(\text{cm1}+\text{cm2})}{2}}{\text{mc} + (1 - \text{mc})\,\frac{(\text{cm1}+\text{cm2})}{2}}$$

2. Male fitness is a function of female fitness and male condition.

$$\text{Malfit[cm1\_, cm2\_, totf\_, gm\_] := totf} * \frac{\text{cm1}^{\text{gm}}}{\text{cm1}^{\text{gm}} + \text{cm2}^{\text{gm}}}$$

Let’s plot some important functions.

The competition function. Blue: g=0, Orange: g=1, Green: g=2

```
Plot[{Malfit[cm1, 1, 1, 0], Malfit[cm1, 1, 1, 1], Malfit[cm1, 1, 1, 2]}, {cm1, 0.01, 2}]
```

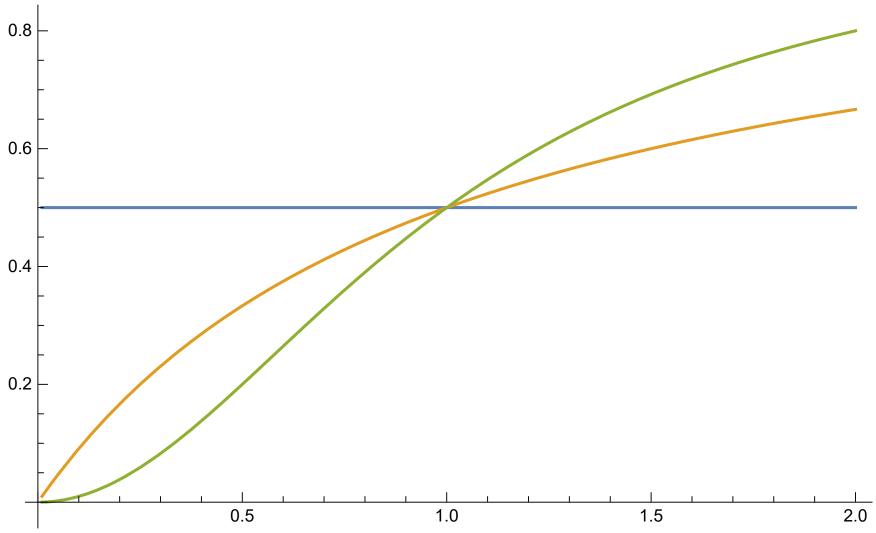

Male fertlization sucess, with Blue: mc=1/100, Orange:mc=1/100, Green: mc=1.

```
Plot[{(2 c)/2 / (1/100 + (1 - (1/100)) (2 c)/2), (2 c)/2 / (1/10 + (1 - (1/10)) (2 c)/2), (2 c)/2 / (1 + (1 - (1)) (2 c)/2)},
 {c, 0, 1}, PlotRange → {0, 1}]
```

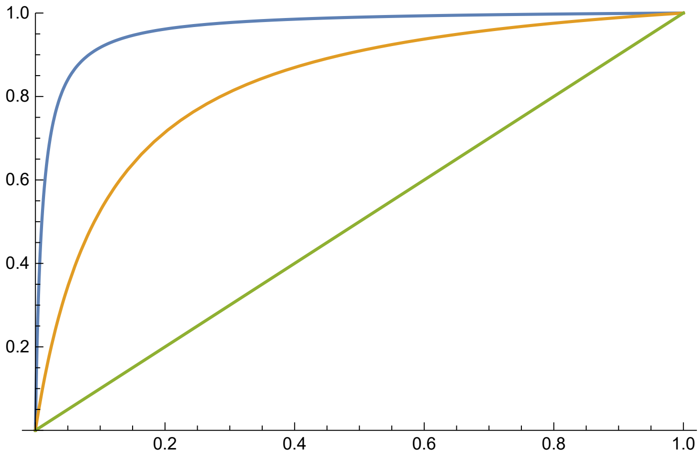

Packaging the model in one function

This function creates a population, runs the fitness assays and spits out the sex-specific variance of the assays ;)

```
popsize = 1000;
sdf = 1/10;
sdm = 1/10;

imf = 0;
mc = 1/100;
gf = 0;
gm = 2;
```

This first function is only used to plot histograms of fitness distribution in the population.

```
JohannaPop[sdf_, sdm_, popsize_, imf_, mc_, gf_, gm_] := (
  malecond = RandomVariate[NormalDistribution[1, sdm], popsize];
  femalecond = RandomVariate[NormalDistribution[1, sdf], popsize];
  Data = Table[{fem1 = Femfit[femalecond[[it * 2 - 1]], femalecond[[it * 2]],
        malecond[[it * 2 - 1]], malecond[[it * 2]], imf, gf, mc];
      fem2 = Femfit[femalecond[[it * 2]], femalecond[[it * 2 - 1]],
        malecond[[it * 2 - 1]], malecond[[it * 2]], imf, gf, mc];
      fem1, fem2, Malfit[malecond[[it * 2 - 1]], malecond[[it * 2]], fem1 + fem2, gm],
      Malfit[malecond[[it * 2]], malecond[[it * 2 - 1]], fem1 + fem2, gm]},
     {it, 1, Length[femalecond] / 2}];
  Females = Union[Transpose[Data][[1]], Transpose[Data][[2]]] /
    Mean[Union[Transpose[Data][[1]], Transpose[Data][[2]]]];
  Males = Union[Transpose[Data][[3]], Transpose[Data][[4]]] /
    Mean[Union[Transpose[Data][[3]], Transpose[Data][[4]]]];
  Return[{Females, Males}])
```

This second function is the main function used to calculate opportunity for selection and plot the main figures.

```
Johanna[sdf_, sdm_, popsize_, imf_, mc_, gf_, gm_] := (
  malecond = RandomVariate[NormalDistribution[1, sdm], popsize];
  femalecond = RandomVariate[NormalDistribution[1, sdf], popsize];
  Data = Table[{fem1 = Femfit[femalecond[[it * 2 - 1]], femalecond[[it * 2]],
       malecond[[it * 2 - 1]], malecond[[it * 2]], imf, gf, mc];
     fem2 = Femfit[femalecond[[it * 2]], femalecond[[it * 2 - 1]],
       malecond[[it * 2 - 1]], malecond[[it * 2]], imf, gf, mc];
     fem1, fem2, Malfit[malecond[[it * 2 - 1]], malecond[[it * 2]], fem1 + fem2, gm],
     Malfit[malecond[[it * 2]], malecond[[it * 2 - 1]], fem1 + fem2, gm]},
    {it, 1, Length[femalecond] / 2}];;
  Varf = Variance[Join[Table[Data[[ö]][[1]], {ö, 1, Length[Data]}],
      Table[Data[[ö]][[2]], {ö, 1, Length[Data]}]]] /
    Mean[Join[Table[Data[[ö]][[1]], {ö, 1, Length[Data]}],
       Table[Data[[ö]][[2]], {ö, 1, Length[Data]}]]]^2;
  Varm = Variance[Join[Table[Data[[ö]][[3]], {ö, 1, Length[Data]}],
      Table[Data[[ö]][[4]], {ö, 1, Length[Data]}]]] /
    Mean[Join[Table[Data[[ö]][[3]], {ö, 1, Length[Data]}],
       Table[Data[[ö]][[4]], {ö, 1, Length[Data]}]]]^2;
  Return[{Varf, Varm}])
```

Results

I. Classical sexual selection. No female competition. Male don't contribute to fecundity.

1st set of assumptions:
- males contribute little to fecundity.
-There is no interaction between male condition and female fitness (no harm, no nuptial gifts).
-Competition in males not in females.
Question: how does sex specific change in variance in condition affects the opp. for selection?

```
popsize = 1000;
imf = 0;
mc = 1 / 100;
gf = 0;
gm = 2;
```

First let's show how male and female distribution translates into fitness distribution. This will be used for Box 1 in the manuscript.

```
popsize = 20 000;
```

```
TableForm[
 {Histogram[{RandomVariate[NormalDistribution[1, 0.1], popsize],
    RandomVariate[NormalDistribution[1, 0.1], popsize]}, {0.05}, ImageSize → 250,
   AspectRatio → 1, Frame → True, FrameLabel → {"Condition", "Individuals"},
   ChartStyle → {Directive[EdgeForm[{Thick, Blue}], FaceForm[{Blue, Opacity[0.15]}]],
     Directive[EdgeForm[{Thick, Orange}], FaceForm[{Orange, Opacity[0.15]}]]},
   BaseStyle → {FaceForm[None], FontSize → 12}, PlotRange → {{0.3, 1.7}, {0, 4000}}],
  Histogram[{RandomVariate[NormalDistribution[1, 0.2], popsize],
    RandomVariate[NormalDistribution[1, 0.1], popsize]}, {0.05}, ImageSize → 250,
   AspectRatio → 1, Frame → True, FrameLabel → {"Condition", "Individuals"},
   ChartStyle → {Directive[EdgeForm[{Thick, Blue}], FaceForm[{Blue, Opacity[0.15]}]],
     Directive[EdgeForm[{Thick, Orange}], FaceForm[{Orange, Opacity[0.15]}]]},
   BaseStyle → {FaceForm[None], FontSize → 12}, PlotRange → {{0.3, 1.7}, {0, 4000}}],
  Histogram[{RandomVariate[NormalDistribution[1, 0.1], popsize],
    RandomVariate[NormalDistribution[1, 0.2], popsize]}, {0.05}, ImageSize → 250,
   AspectRatio → 1, Frame → True, FrameLabel → {"Condition", "Individuals"},
   ChartStyle → {Directive[EdgeForm[{Thick, Blue}], FaceForm[{Blue, Opacity[0.15]}]],
     Directive[EdgeForm[{Thick, Orange}], FaceForm[{Orange, Opacity[0.15]}]]},
   BaseStyle → {FaceForm[None], FontSize → 12}, PlotRange → {{0.3, 1.7}, {0, 4000}}]
 }]
```

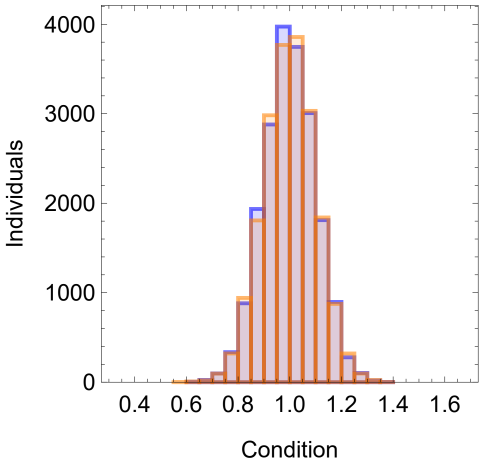
Individuals
Condition

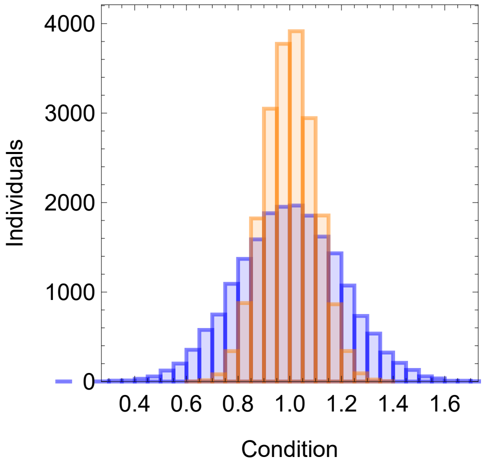
Individuals
Condition

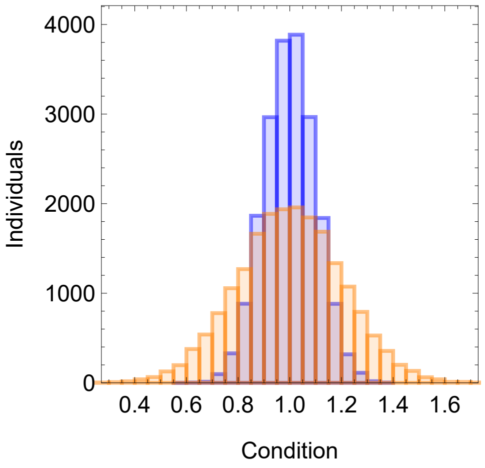
Individuals
Condition

```
TableForm[
 {Histogram[JohannaPop[0.1, 0.1, 2000, imf, mc, gf, gm],
   {0.05}, ImageSize → 250, AspectRatio → 1, Frame → True,
   FrameLabel → {"Relative Fitness", "Individuals"}, ChartStyle → {Directive[
      EdgeForm[{Thickness[0.015], Blue}], FaceForm[{Blue, Opacity[0.25]}]], Directive[
      EdgeForm[{Thickness[0.015], Orange}], FaceForm[{Orange, Opacity[0.25]}]]},
   BaseStyle → {FaceForm[None], FontSize → 12}, PlotRange → {{0.3, 1.7}, {0, 400}}],
  Histogram[JohannaPop[0.2, 0.1, 2000, imf, mc, gf, gm], {0.05}, ImageSize → 250,
   AspectRatio → 1, Frame → True, FrameLabel → {"Relative Fitness", "Individuals"},
   ChartStyle → {Directive[EdgeForm[{Thick, Blue}], FaceForm[{Blue, Opacity[0.15]}]],
     Directive[EdgeForm[{Thick, Orange}], FaceForm[{Orange, Opacity[0.15]}]]},
   BaseStyle → {FaceForm[None], FontSize → 12}, PlotRange → {{0.3, 1.7}, {0, 400}}],
  Histogram[JohannaPop[0.1, 0.2, 2000, imf, mc, gf, gm], {0.05}, ImageSize → 250,
   AspectRatio → 1, Frame → True, FrameLabel → {"Relative Fitness", "Individuals"},
   ChartStyle → {Directive[EdgeForm[{Thick, Blue}], FaceForm[{Blue, Opacity[0.15]}]],
     Directive[EdgeForm[{Thick, Orange}], FaceForm[{Orange, Opacity[0.15]}]]},
   BaseStyle → {FaceForm[None], FontSize → 12}, PlotRange → {{0.3, 1.7}, {0, 400}}]
 }]
```

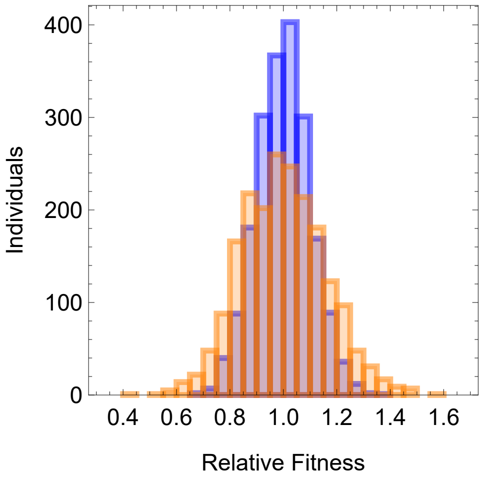

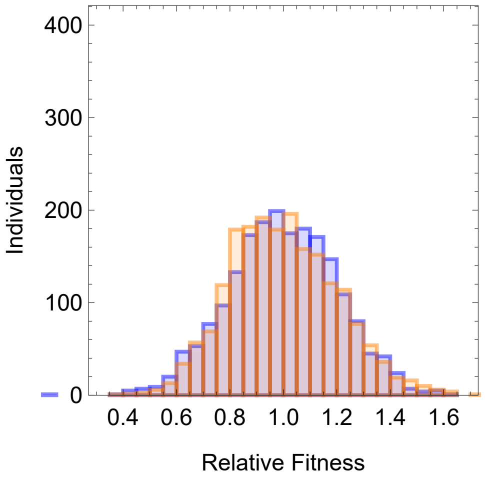

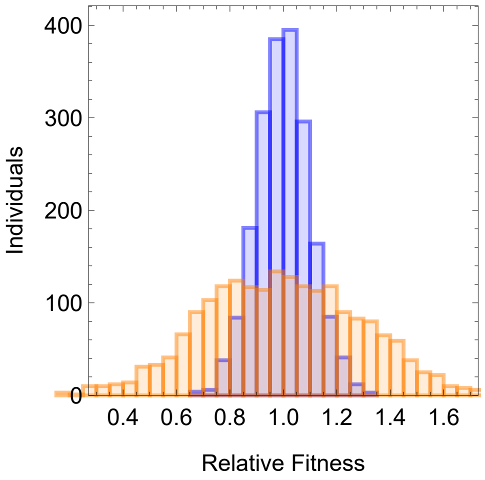

Plot male and female variance separately

```
DatF1 = Table[Table[Datt = Johanna[sdchange, sdchange, popsize, imf, mc, gf, gm];
     {sdchange, Datt[[1]]}, {sdchange, {0.1, 0.2, 0.3, 0.4}}], {num, 0, 10}];
DatM1 = Table[Table[Datt = Johanna[sdchange, sdchange, popsize, imf, mc, gf, gm];
     {sdchange, Datt[[2]]}, {sdchange, {0.1, 0.2, 0.3, 0.4}}], {num, 0, 10}];
DatBF1 = Table[Table[Datt = Johanna[sdchange, sdm, popsize, imf, mc, gf, gm];
     {sdchange, Datt[[1]]}, {sdchange, {0.1, 0.2, 0.3, 0.4}}], {num, 0, 10}];
DatBM1 = Table[Table[Datt = Johanna[sdchange, sdm, popsize, imf, mc, gf, gm];
     {sdchange, Datt[[2]]}, {sdchange, {0.1, 0.2, 0.3, 0.4}}], {num, 0, 10}];
DatCF1 = Table[Table[Datt = Johanna[sdf, sdchange, popsize, imf, mc, gf, gm];
     {sdchange, Datt[[1]]}, {sdchange, {0.1, 0.2, 0.3, 0.4}}], {num, 0, 10}];
DatCM1 = Table[Table[Datt = Johanna[sdf, sdchange, popsize, imf, mc, gf, gm];
     {sdchange, Datt[[2]]}, {sdchange, {0.1, 0.2, 0.3, 0.4}}], {num, 0, 10}];

AF = ListLinePlot[DatF1, PlotRange → All, PlotStyle → Directive[Black, Opacity[0.3]]];
AFmean = ListLinePlot[Mean[DatF1], PlotRange → All,
   PlotMarkers → {"◆", 30}, PlotStyle → Directive[Black]];
AM = ListLinePlot[DatM1, PlotRange → All, PlotStyle → Directive[Black, Opacity[0.3]]];
AMmean = ListLinePlot[Mean[DatM1], PlotRange → All,
   PlotMarkers → {"●", 30}, PlotStyle → Directive[Black]];
ABF = ListLinePlot[DatBF1, PlotRange → All,
   PlotStyle → Directive[Purple, Opacity[0.3]]];
ABFmean = ListLinePlot[Mean[DatBF1], PlotRange → All,
   PlotMarkers → {"◆", 30}, PlotStyle → Directive[Purple]];
ABM = ListLinePlot[DatBM1, PlotRange → All,
   PlotStyle → Directive[Purple, Opacity[0.3]]];
ABMmean = ListLinePlot[Mean[DatBM1], PlotRange → All,
   PlotMarkers → {"●", 30}, PlotStyle → Directive[Purple]];
ACF = ListLinePlot[DatCF1, PlotRange → All, PlotStyle → Directive[Brown, Opacity[0.3]]];
ACFmean = ListLinePlot[Mean[DatCF1], PlotRange → All,
   PlotMarkers → {"◆", 30}, PlotStyle → Directive[Brown]];
ACM = ListLinePlot[DatCM1, PlotRange → All, PlotStyle → Directive[Brown, Opacity[0.3]]];
ACMmean = ListLinePlot[Mean[DatCM1], PlotRange → All,
   PlotMarkers → {"●", 30}, PlotStyle → Directive[Brown]];
```

```
GraphicsRow[{
  Show[AM, AF, AFmean, AMmean, ImageSize → 450, PlotRange -> All,
   BaseStyle → {FontSize → 15}, PlotLabel → "a. Both sexes are stressed",
   Axes → False, Frame → {{True, False}, {True, False}},
   FrameLabel → {{ "Im If", None}, {"Stress", None}}, LabelStyle -> Directive[Black]],
  Show[ABM, ABF, ABMmean, ABFmean, ImageSize → 450, PlotRange -> All,
   BaseStyle → {FontSize → 15}, PlotLabel → "b. Females only are stressed",
   Axes → False, Frame → {{True, False}, {True, False}},
   FrameLabel → {{ None, None}, {"Stress", None}}, LabelStyle -> Directive[Black]],
  Show[ACM, ACF, ACMmean, ACFmean, ImageSize → 450, PlotRange -> All,
   BaseStyle → {FontSize → 15}, PlotLabel → "c. Males only are stressed",
   Axes → False, Frame → {{True, False}, {True, False}}, FrameLabel →
    {{ None, None}, {"Stress", None}}, LabelStyle -> Directive[Black]]}, Spacings → 1]
```

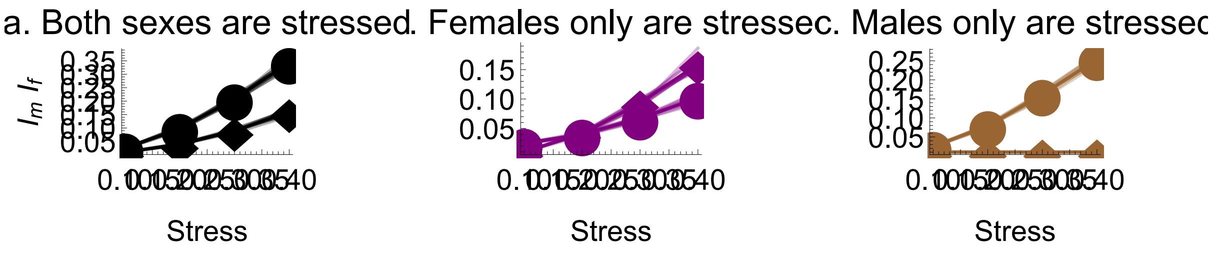


```
DatA1 = Table[Table[Datat = Johanna[sdchange, sdchange, popsize, imf, mc, gf, gm];
    Log[Datat[[2]] / Datat[[1]]], {sdchange, {0.1, 0.2, 0.3, 0.4}}], {num, 0, 20}];
DatB21 = Table[Table[Datat = Johanna[sdchange, sdm, popsize, imf, mc, gf, gm];
    Log[Datat[[2]] / Datat[[1]]], {sdchange, {0.1, 0.2, 0.3, 0.4}}], {num, 0, 20}];
DatB1 = Table[Table[Datat = Johanna[sdf, sdchange, popsize, imf, mc, gf, gm];
    Log[Datat[[2]] / Datat[[1]]], {sdchange, {0.1, 0.2, 0.3, 0.4}}], {num, 0, 20}];
A1 = ListLinePlot[DatA1, PlotRange → All, PlotStyle → Directive[Black, Opacity[0.3]]];
B1 = ListLinePlot[DatB1,
   PlotRange → {{1, 4}, {Min[0, Min[DatB21, DatA1, DatB1]], Max[DatB21, DatA1, DatB1]}},
   PlotStyle → Directive[Brown, Opacity[0.3]]];
B21 = ListLinePlot[DatB21, PlotStyle →
    Directive[Purple, PlotRange → All, Opacity[0.3]]];
A31 = ListLinePlot[Table[Mean[Transpose[DatA1][[ä]]], {ä, 1, 4}],
   PlotStyle → Directive[Black, Thickness[0.008]], PlotRange → All];
B31 = ListLinePlot[Table[Mean[Transpose[DatB1][[ä]]], {ä, 1, 4}],
   PlotStyle → Directive[Brown, Thickness[0.008]], PlotRange → All];
B41 = ListLinePlot[Table[Mean[Transpose[DatB21][[ä]]], {ä, 1, 4}],
   PlotStyle → Directive[Purple, Thickness[0.008]], PlotRange → All];
```

```
Show[B1, A1, B21, A31, B31, B41, ImageSize → 650,
 PlotRange -> All, BaseStyle → {FontSize → 15}, PlotLabel → "",
 Axes → False, Frame → {{True, False}, {True, False}},
 FrameLabel → {{"Log (Im/If)", None}, {"Stress", None}},
 LabelStyle -> Directive[Black]]
```

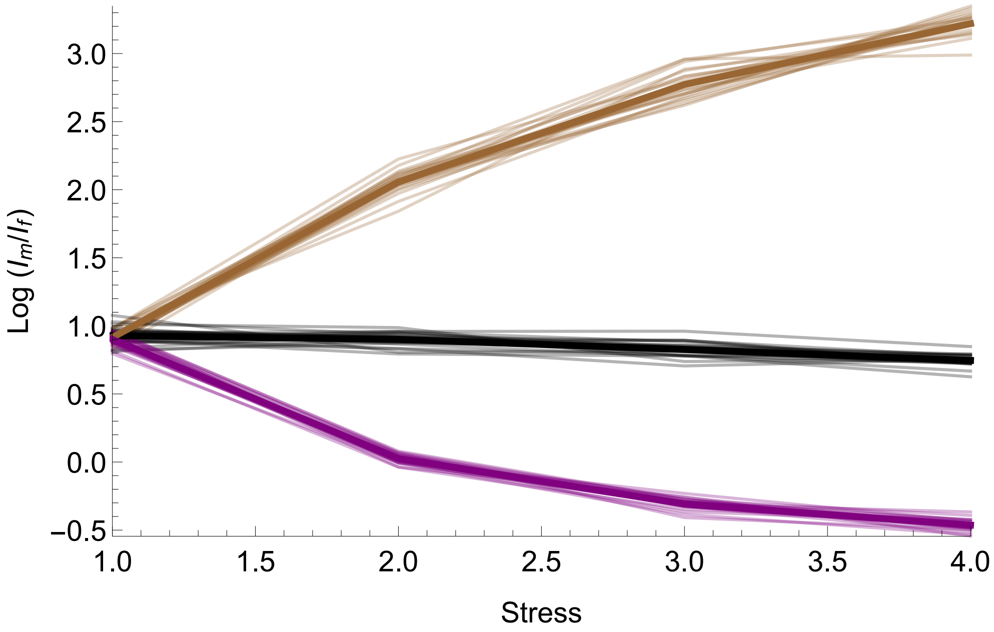

Results:

II. Classical sexual selection+Nuptial gifts

2nd set of assumptions:

- males contribute little to fecundity.

-There are condition dependent nuptial gifts!

-Slight environmental stress, with no sex specificity.

-Competition in males not in females.

Question: how does sex specific change in variance in condition affects the opp. for selection?

```
sdm = 1/10;
sdf = 1/10;
popsize = 1000;

imf = 5/10;
mc = 1/100;
gf = 0;
gm = 2;
```

Redifining the graphic style of the basic male competition scenario to use as comparison (smaller markers, red.)

```
diam = Graphics[{EdgeForm[{Black, Thin}],
    FaceForm[Red], Polygon[{{-1, 0}, {0, 1}, {1, 0}, {0, -1}}]}];
circ = Graphics[{EdgeForm[{Black, Thin}], FaceForm[Red], Disk[]}];
```

```
AFmean = ListLinePlot[Mean[DatF1], PlotRange → All, PlotMarkers → {diam, 0.05}];
AMmean = ListLinePlot[Mean[DatM1], PlotRange → All, PlotMarkers → {circ, 0.05}];
ABFmean = ListLinePlot[Mean[DatBF1], PlotRange → All, PlotMarkers → {diam, 0.05}];
ABMmean = ListLinePlot[Mean[DatBM1], PlotRange → All, PlotMarkers → {circ, 0.05}];
ACFmean = ListLinePlot[Mean[DatCF1], PlotRange → All, PlotMarkers → {diam, 0.05}];
ACMmean = ListLinePlot[Mean[DatCM1], PlotRange → All, PlotMarkers → {circ, 0.05}];
```

```
DatF = Table[Table[Datt = Johanna[sdchange, sdchange, popsize, imf, mc, gf, gm];
     {sdchange, Datt[[1]]}, {sdchange, {0.1, 0.2, 0.3, 0.4}}], {num, 0, 10}];
DatM = Table[Table[Datt = Johanna[sdchange, sdchange, popsize, imf, mc, gf, gm];
     {sdchange, Datt[[2]]}, {sdchange, {0.1, 0.2, 0.3, 0.4}}], {num, 0, 10}];
DatBF = Table[Table[Datt = Johanna[sdchange, sdm, popsize, imf, mc, gf, gm];
     {sdchange, Datt[[1]]}, {sdchange, {0.1, 0.2, 0.3, 0.4}}], {num, 0, 10}];
DatBM = Table[Table[Datt = Johanna[sdchange, sdm, popsize, imf, mc, gf, gm];
     {sdchange, Datt[[2]]}, {sdchange, {0.1, 0.2, 0.3, 0.4}}], {num, 0, 10}];
DatCF = Table[Table[Datt = Johanna[sdf, sdchange, popsize, imf, mc, gf, gm];
     {sdchange, Datt[[1]]}, {sdchange, {0.1, 0.2, 0.3, 0.4}}], {num, 0, 10}];
DatCM = Table[Table[Datt = Johanna[sdf, sdchange, popsize, imf, mc, gf, gm];
     {sdchange, Datt[[2]]}, {sdchange, {0.1, 0.2, 0.3, 0.4}}], {num, 0, 10}];
```

```
AF = ListLinePlot[DatF, PlotRange → All, PlotStyle → Directive[Black, Opacity[0.3]]];
AFmean2 = ListLinePlot[Mean[DatF], PlotRange → All,
   PlotMarkers → {"◆", 30}, PlotStyle → Directive[Black]];
AM = ListLinePlot[DatM, PlotRange → All, PlotStyle → Directive[Black, Opacity[0.3]]];
AMmean2 = ListLinePlot[Mean[DatM], PlotRange → All,
   PlotMarkers → {"●", 30}, PlotStyle → Directive[Black]];
ABF = ListLinePlot[DatBF, PlotRange → All, PlotStyle → Directive[Purple, Opacity[0.3]]];
ABFmean2 = ListLinePlot[Mean[DatBF], PlotRange → All,
   PlotMarkers → {"◆", 30}, PlotStyle → Directive[Purple]];
ABM = ListLinePlot[DatBM, PlotRange → All, PlotStyle → Directive[Purple, Opacity[0.3]]];
ABMmean2 = ListLinePlot[Mean[DatBM], PlotRange → All,
   PlotMarkers → {"●", 30}, PlotStyle → Directive[Purple]];
ACF = ListLinePlot[DatCF, PlotRange → All, PlotStyle → Directive[Brown, Opacity[0.3]]];
ACFmean2 = ListLinePlot[Mean[DatCF], PlotRange → All,
   PlotMarkers → {"◆", 30}, PlotStyle → Directive[Brown]];
ACM = ListLinePlot[DatCM, PlotRange → All, PlotStyle → Directive[Brown, Opacity[0.3]]];
ACMmean2 = ListLinePlot[Mean[DatCM], PlotRange → All,
   PlotMarkers → {"●", 30}, PlotStyle → Directive[Brown]];
```

Red markers will show the original scenario for comparison.

```
GraphicsRow[{
  Show[AM, AF, AFmean2, AMmean2, AFmean, AMmean, ImageSize → 450, PlotRange -> All,
   BaseStyle → {FontSize → 15}, PlotLabel → "a. Both sexes are stressed",
   Axes → False, Frame → {{True, False}, {True, False}},
   FrameLabel → {{"Im If", None}, {"Stress", None}}, LabelStyle -> Directive[Black]],
  Show[ABM, ABF, ABMmean2, ABFmean2, ABMmean, ABFmean, ImageSize → 450, PlotRange -> All,
   BaseStyle → {FontSize → 15}, PlotLabel → "b. Females only are stressed",
   Axes → False, Frame → {{True, False}, {True, False}},
   FrameLabel → {{None, None}, {"Stress", None}}, LabelStyle -> Directive[Black]],
  Show[ACM, ACF, ACMmean2, ACFmean2, ACMmean, ACFmean, ImageSize → 450, PlotRange -> All,
   BaseStyle → {FontSize → 15}, PlotLabel → "c. Males only are stressed",
   Axes → False, Frame → {{True, False}, {True, False}}, FrameLabel →
    {{None, None}, {"Stress", None}}, LabelStyle -> Directive[Black]]}, Spacings → 1]
```

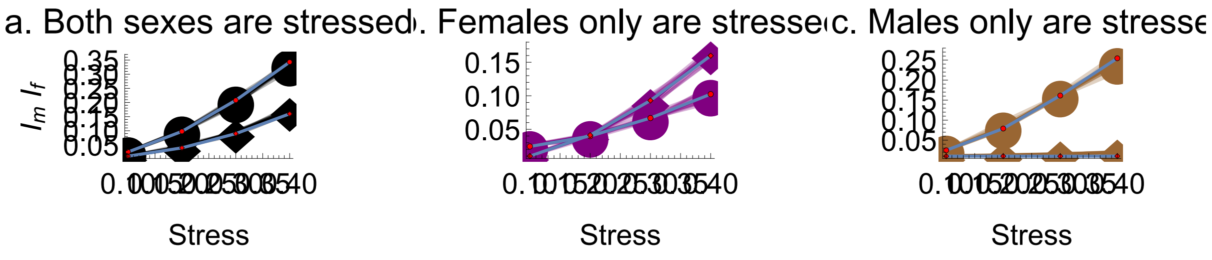

Redifine graphic style or reference scenario for the log ratio

```
A31 = ListLinePlot[Table[Mean[Transpose[DatA1][[ä]]], {ä, 1, 4}],
   PlotStyle → Directive[Black, Thickness[0.005], Dashed], PlotRange → All];
B31 = ListLinePlot[Table[Mean[Transpose[DatB1][[ä]]], {ä, 1, 4}],
   PlotStyle → Directive[Brown, Thickness[0.005], Dashed], PlotRange → All];
B41 = ListLinePlot[Table[Mean[Transpose[DatB21][[ä]]], {ä, 1, 4}],
   PlotStyle → Directive[Purple, Thickness[0.005], Dashed], PlotRange → All];
```

```
DatA = Table[Table[Datat = Johanna[sdchange, sdchange, popsize, imf, mc, gf, gm];
    Log[Datat[[2]] / Datat[[1]]], {sdchange, {0.1, 0.2, 0.3, 0.4}}], {num, 0, 20}];
DatB2 = Table[Table[Datat = Johanna[sdchange, sdm, popsize, imf, mc, gf, gm];
    Log[Datat[[2]] / Datat[[1]]], {sdchange, {0.1, 0.2, 0.3, 0.4}}], {num, 0, 20}];
DatB = Table[Table[Datat = Johanna[sdf, sdchange, popsize, imf, mc, gf, gm];
    Log[Datat[[2]] / Datat[[1]]], {sdchange, {0.1, 0.2, 0.3, 0.4}}], {num, 0, 20}];
Ab = ListLinePlot[DatA, PlotRange → All, PlotStyle → Directive[Black, Opacity[0.3]]];
Bb = ListLinePlot[DatB,
   PlotRange → {{1, 4}, {Min[0, Min[DatB2, DatA, DatB]], Max[DatB2, DatA, DatB]}},
   PlotStyle → Directive[Brown, Opacity[0.3]]];
B2b = ListLinePlot[DatB2, PlotStyle → Directive[Purple, PlotRange → All, Opacity[0.3]]];
A3b = ListLinePlot[Table[Mean[Transpose[DatA][[ä]]], {ä, 1, 4}],
   PlotStyle → Directive[Black, Thickness[0.005], Opacity[0.7]], PlotRange → All];
B3b = ListLinePlot[Table[Mean[Transpose[DatB][[ä]]], {ä, 1, 4}],
   PlotStyle → Directive[Brown, Thickness[0.005], Opacity[0.7]], PlotRange → All];
B4b = ListLinePlot[Table[Mean[Transpose[DatB2][[ä]]], {ä, 1, 4}],
   PlotStyle → Directive[Purple, Thickness[0.005], Opacity[0.7]], PlotRange → All];
```

Dashed lines will show the original scenario for comparison.

```
Show[Bb, Ab, B2b, A3b, B3b, B4b, A31, B31, B41,
 ImageSize → 650, PlotRange -> All, BaseStyle → {FontSize → 15},
 PlotLabel → "", Axes → False, Frame → {{True, False}, {True, False}},
 FrameLabel → {{"Log (Im/If)", None}, {"Stress", None}},
 LabelStyle -> Directive[Black]]
```

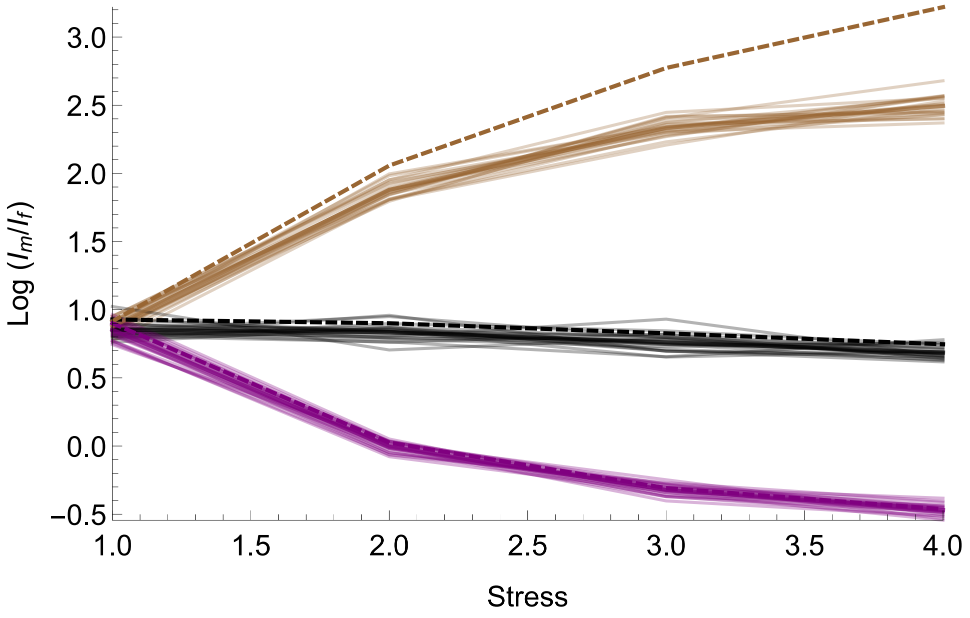

Result: The difference in the male-limited stress part is due to the fact tha female opp for sel increases with stress now too, as a result of the nuptial gift.

III. Classical sexual selection with male harm

3rd set of assumptions:
- males contribute little to fecundity.
-There is condition dependent male harm!
-Slight environmental stress, with no sex specificity.
-Competition in males not in females.
Question: how does sex specific change in variance in condition affects the opp. for selection?

```
sdf = 1/10;
sdm = 1/10;
popsize = 1000;
imf = -5/10;
mc = 1/100;
gf = 0;
gm = 2;
```

```
DatF = Table[Table[Datt = Johanna[sdchange, sdchange, popsize, imf, mc, gf, gm];
    {sdchange, Datt[[1]]}, {sdchange, {0.1, 0.2, 0.3, 0.4}}], {num, 0, 10}];
DatM = Table[Table[Datt = Johanna[sdchange, sdchange, popsize, imf, mc, gf, gm];
    {sdchange, Datt[[2]]}, {sdchange, {0.1, 0.2, 0.3, 0.4}}], {num, 0, 10}];
DatBF = Table[Table[Datt = Johanna[sdchange, sdm, popsize, imf, mc, gf, gm];
    {sdchange, Datt[[1]]}, {sdchange, {0.1, 0.2, 0.3, 0.4}}], {num, 0, 10}];
DatBM = Table[Table[Datt = Johanna[sdchange, sdm, popsize, imf, mc, gf, gm];
    {sdchange, Datt[[2]]}, {sdchange, {0.1, 0.2, 0.3, 0.4}}], {num, 0, 10}];
DatCF = Table[Table[Datt = Johanna[sdf, sdchange, popsize, imf, mc, gf, gm];
    {sdchange, Datt[[1]]}, {sdchange, {0.1, 0.2, 0.3, 0.4}}], {num, 0, 10}];
DatCM = Table[Table[Datt = Johanna[sdf, sdchange, popsize, imf, mc, gf, gm];
    {sdchange, Datt[[2]]}, {sdchange, {0.1, 0.2, 0.3, 0.4}}], {num, 0, 10}];

AF = ListLinePlot[DatF, PlotRange → All, PlotStyle → Directive[Black, Opacity[0.3]]];
AFmean2 = ListLinePlot[Mean[DatF], PlotRange → All,
   PlotMarkers → {"◆", 30}, PlotStyle → Directive[Black]];
AM = ListLinePlot[DatM, PlotRange → All, PlotStyle → Directive[Black, Opacity[0.3]]];
AMmean2 = ListLinePlot[Mean[DatM], PlotRange → All,
   PlotMarkers → {"●", 30}, PlotStyle → Directive[Black]];
ABF = ListLinePlot[DatBF, PlotRange → All, PlotStyle → Directive[Purple, Opacity[0.3]]];
ABFmean2 = ListLinePlot[Mean[DatBF], PlotRange → All,
   PlotMarkers → {"◆", 30}, PlotStyle → Directive[Purple]];
ABM = ListLinePlot[DatBM, PlotRange → All, PlotStyle → Directive[Purple, Opacity[0.3]]];
ABMmean2 = ListLinePlot[Mean[DatBM], PlotRange → All,
   PlotMarkers → {"●", 30}, PlotStyle → Directive[Purple]];
ACF = ListLinePlot[DatCF, PlotRange → All, PlotStyle → Directive[Brown, Opacity[0.3]]];
ACFmean2 = ListLinePlot[Mean[DatCF], PlotRange → All,
   PlotMarkers → {"◆", 30}, PlotStyle → Directive[Brown]];
ACM = ListLinePlot[DatCM, PlotRange → All, PlotStyle → Directive[Brown, Opacity[0.3]]];
ACMmean2 = ListLinePlot[Mean[DatCM], PlotRange → All,
   PlotMarkers → {"●", 30}, PlotStyle → Directive[Brown]];

GraphicsRow[{
  Show[AM, AF, AFmean2, AMmean2, AFmean, AMmean, ImageSize → 450, PlotRange -> All,
   BaseStyle → {FontSize → 15}, PlotLabel → "a. Both sexes are stressed",
   Axes → False, Frame → {{True, False}, {True, False}},
   FrameLabel → {{"I_m I_f", None}, {"Stress", None}}, LabelStyle -> Directive[Black]],
  Show[ABM, ABF, ABMmean2, ABFmean2, ABMmean, ABFmean, ImageSize → 450, PlotRange -> All,
   BaseStyle → {FontSize → 15}, PlotLabel → "b. Females only are stressed",
   Axes → False, Frame → {{True, False}, {True, False}},
   FrameLabel → {{None, None}, {"Stress", None}}, LabelStyle -> Directive[Black]],
  Show[ACM, ACF, ACMmean2, ACFmean2, ACMmean, ACFmean, ImageSize → 450, PlotRange -> All,
   BaseStyle → {FontSize → 15}, PlotLabel → "c. Males only are stressed",
   Axes → False, Frame → {{True, False}, {True, False}}, FrameLabel →
    {{None, None}, {"Stress", None}}, LabelStyle -> Directive[Black]]}, Spacings → 1]
```

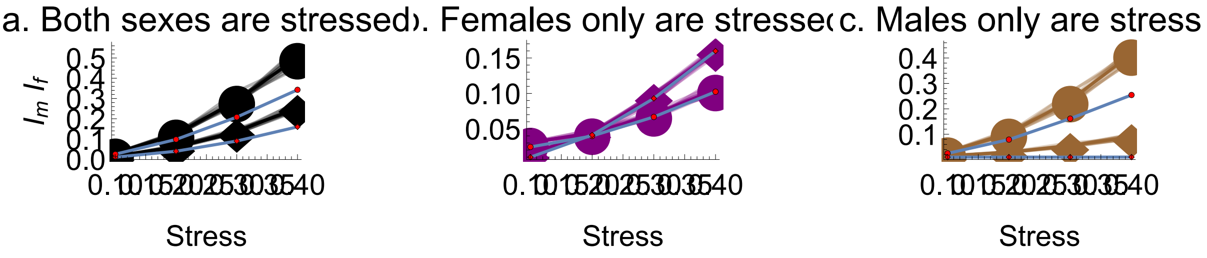

```
DatA = Table[Table[Datat = Johanna[sdchange, sdchange, popsize, imf, mc, gf, gm];
     Log[Datat[[2]] / Datat[[1]]], {sdchange, {0.1, 0.2, 0.3, 0.4}}], {num, 0, 10}];
DatB2 = Table[Table[Datat = Johanna[sdchange, sdm, popsize, imf, mc, gf, gm];
     Log[Datat[[2]] / Datat[[1]]], {sdchange, {0.1, 0.2, 0.3, 0.4}}], {num, 0, 10}];
DatB = Table[Table[Datat = Johanna[sdf, sdchange, popsize, imf, mc, gf, gm];
     Log[Datat[[2]] / Datat[[1]]], {sdchange, {0.1, 0.2, 0.3, 0.4}}], {num, 0, 10}];
Ac = ListLinePlot[DatA, PlotRange → All, PlotStyle → Directive[Black, Opacity[0.3]]];
Bc = ListLinePlot[DatB,
   PlotRange → {{1, 4}, {Min[0, Min[DatB2, DatA, DatB]], Max[DatB2, DatA, DatB]}},
   PlotStyle → Directive[Brown, Opacity[0.3]]];
B2c = ListLinePlot[DatB2, PlotStyle → Directive[Purple, PlotRange → All, Opacity[0.3]]];
A3c = ListLinePlot[Table[Mean[Transpose[DatA][[ä]]], {ä, 1, 4}],
   PlotStyle → Directive[Black, Thickness[0.005], Opacity[0.7]], PlotRange → All];
B3c = ListLinePlot[Table[Mean[Transpose[DatB][[ä]]], {ä, 1, 4}],
   PlotStyle → Directive[Brown, Thickness[0.005], Opacity[0.7]], PlotRange → All];
B4c = ListLinePlot[Table[Mean[Transpose[DatB2][[ä]]], {ä, 1, 4}],
   PlotStyle → Directive[Purple, Thickness[0.005], Opacity[0.7]], PlotRange → All];
```

```
Show[Bc, Ac, B2c, A3c, B3c, B4c, A31, B31, B41,
 ImageSize → 650, PlotRange -> All, BaseStyle → {FontSize → 15},
 PlotLabel → "", Axes → False, Frame → {{True, False}, {True, False}},
 FrameLabel → {{"Log (Im/If)", None}, {"Stress", None}},
 LabelStyle -> Directive[Black]]
```

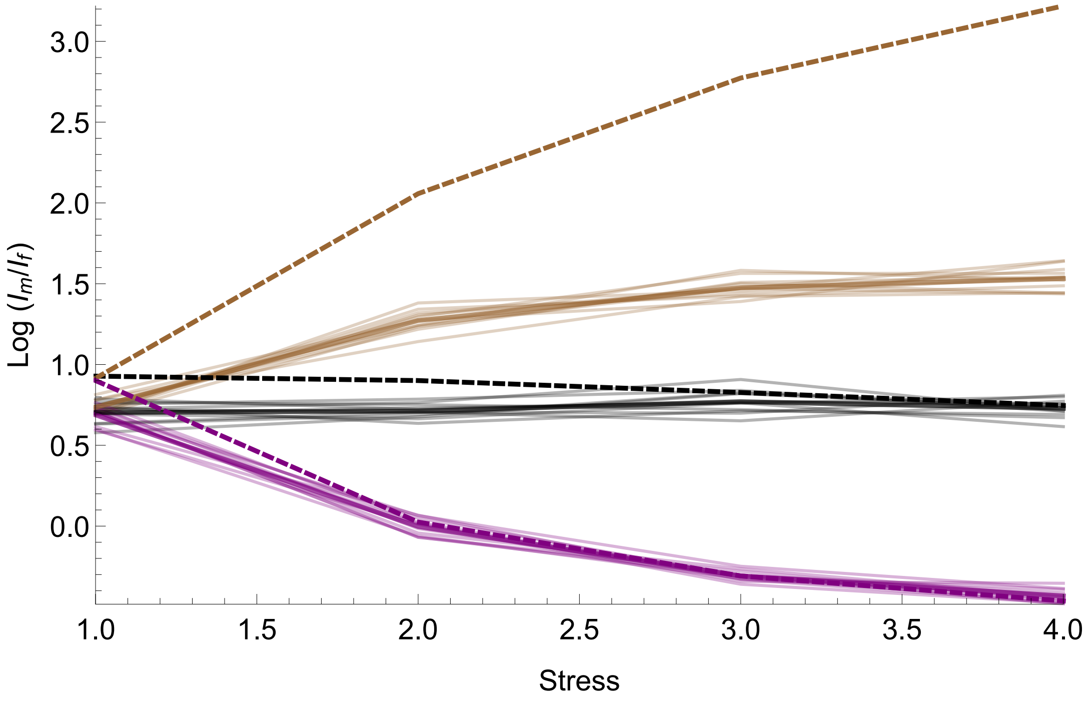

III.b Classical sexual selection with sperm limitation

3rd set of assumptions:

- males contribute little to fecundity.

-There is condition dependent male harm!

-Slight environmental stress, with no sex specificity.

-Competition in males not in females.

Question: how does sex specific change in variance in condition affects the opp. for selection?

```
sdf = 1/10;
sdm = 1/10;
popsize = 1000;
imf = 0;
mc = 1/5;
gf = 0;
gm = 2;
```

```
DatF = Table[Table[Datt = Johanna[sdchange, sdchange, popsize, imf, mc, gf, gm];
    {sdchange, Datt[[1]]}, {sdchange, {0.1, 0.2, 0.3, 0.4}}], {num, 0, 10}];
DatM = Table[Table[Datt = Johanna[sdchange, sdchange, popsize, imf, mc, gf, gm];
    {sdchange, Datt[[2]]}, {sdchange, {0.1, 0.2, 0.3, 0.4}}], {num, 0, 10}];
DatBF = Table[Table[Datt = Johanna[sdchange, sdm, popsize, imf, mc, gf, gm];
    {sdchange, Datt[[1]]}, {sdchange, {0.1, 0.2, 0.3, 0.4}}], {num, 0, 10}];
DatBM = Table[Table[Datt = Johanna[sdchange, sdm, popsize, imf, mc, gf, gm];
    {sdchange, Datt[[2]]}, {sdchange, {0.1, 0.2, 0.3, 0.4}}], {num, 0, 10}];
DatCF = Table[Table[Datt = Johanna[sdf, sdchange, popsize, imf, mc, gf, gm];
    {sdchange, Datt[[1]]}, {sdchange, {0.1, 0.2, 0.3, 0.4}}], {num, 0, 10}];
DatCM = Table[Table[Datt = Johanna[sdf, sdchange, popsize, imf, mc, gf, gm];
    {sdchange, Datt[[2]]}, {sdchange, {0.1, 0.2, 0.3, 0.4}}], {num, 0, 10}];
```

```
AF = ListLinePlot[DatF, PlotRange → All, PlotStyle → Directive[Black, Opacity[0.3]]];
AFmean2 = ListLinePlot[Mean[DatF], PlotRange → All,
   PlotMarkers → {"◆", 30}, PlotStyle → Directive[Black]];
AM = ListLinePlot[DatM, PlotRange → All, PlotStyle → Directive[Black, Opacity[0.3]]];
AMmean2 = ListLinePlot[Mean[DatM], PlotRange → All,
   PlotMarkers → {"●", 30}, PlotStyle → Directive[Black]];
ABF = ListLinePlot[DatBF, PlotRange → All, PlotStyle → Directive[Purple, Opacity[0.3]]];
ABFmean2 = ListLinePlot[Mean[DatBF], PlotRange → All,
   PlotMarkers → {"◆", 30}, PlotStyle → Directive[Purple]];
ABM = ListLinePlot[DatBM, PlotRange → All, PlotStyle → Directive[Purple, Opacity[0.3]]];
ABMmean2 = ListLinePlot[Mean[DatBM], PlotRange → All,
   PlotMarkers → {"●", 30}, PlotStyle → Directive[Purple]];
ACF = ListLinePlot[DatCF, PlotRange → All, PlotStyle → Directive[Brown, Opacity[0.3]]];
ACFmean2 = ListLinePlot[Mean[DatCF], PlotRange → All,
   PlotMarkers → {"◆", 30}, PlotStyle → Directive[Brown]];
ACM = ListLinePlot[DatCM, PlotRange → All, PlotStyle → Directive[Brown, Opacity[0.3]]];
ACMmean2 = ListLinePlot[Mean[DatCM], PlotRange → All,
   PlotMarkers → {"●", 30}, PlotStyle → Directive[Brown]];
```

```
GraphicsRow[{
  Show[AM, AF, AFmean2, AMmean2, AFmean, AMmean, ImageSize → 450, PlotRange -> All,
   BaseStyle → {FontSize → 15}, PlotLabel → "a. Both sexes are stressed",
   Axes → False, Frame → {{True, False}, {True, False}},
   FrameLabel → {{ "Im If", None}, {"Stress", None}}, LabelStyle -> Directive[Black]],
  Show[ABM, ABF, ABMmean2, ABFmean2, ABMmean, ABFmean, ImageSize → 450, PlotRange -> All,
   BaseStyle → {FontSize → 15}, PlotLabel → "b. Females only are stressed",
   Axes → False, Frame → {{True, False}, {True, False}},
   FrameLabel → {{ None, None}, {"Stress", None}}, LabelStyle -> Directive[Black]],
  Show[ACM, ACF, ACMmean2, ACFmean2, ACMmean, ACFmean, ImageSize → 450, PlotRange -> All,
   BaseStyle → {FontSize → 15}, PlotLabel → "c. Males only are stressed",
   Axes → False, Frame → {{True, False}, {True, False}}, FrameLabel →
    {{ None, None}, {"Stress", None}}, LabelStyle -> Directive[Black]]}, Spacings → 1]
```

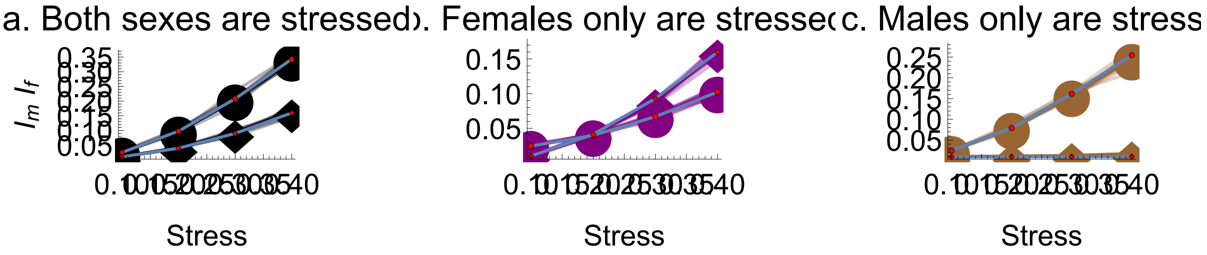


```
DatA = Table[Table[Datat = Johanna[sdchange, sdchange, popsize, imf, mc, gf, gm];
    Log[Datat[[2]] / Datat[[1]]], {sdchange, {0.1, 0.2, 0.3, 0.4}}], {num, 0, 10}];
DatB2 = Table[Table[Datat = Johanna[sdchange, sdm, popsize, imf, mc, gf, gm];
    Log[Datat[[2]] / Datat[[1]]], {sdchange, {0.1, 0.2, 0.3, 0.4}}], {num, 0, 10}];
DatB = Table[Table[Datat = Johanna[sdf, sdchange, popsize, imf, mc, gf, gm];
    Log[Datat[[2]] / Datat[[1]]], {sdchange, {0.1, 0.2, 0.3, 0.4}}], {num, 0, 10}];
Ac = ListLinePlot[DatA, PlotRange → All, PlotStyle → Directive[Black, Opacity[0.3]]];
Bc = ListLinePlot[DatB,
   PlotRange → {{1, 4}, {Min[0, Min[DatB2, DatA, DatB]], Max[DatB2, DatA, DatB]}},
   PlotStyle → Directive[Brown, Opacity[0.3]]];
B2c = ListLinePlot[DatB2, PlotStyle → Directive[Purple, PlotRange → All, Opacity[0.3]]];
A3c = ListLinePlot[Table[Mean[Transpose[DatA][[ä]]], {ä, 1, 4}],
   PlotStyle → Directive[Black, Thickness[0.005], Opacity[0.7]], PlotRange → All];
B3c = ListLinePlot[Table[Mean[Transpose[DatB][[ä]]], {ä, 1, 4}],
   PlotStyle → Directive[Brown, Thickness[0.005], Opacity[0.7]], PlotRange → All];
B4c = ListLinePlot[Table[Mean[Transpose[DatB2][[ä]]], {ä, 1, 4}],
   PlotStyle → Directive[Purple, Thickness[0.005], Opacity[0.7]], PlotRange → All];
```

```
Show[Bc, Ac, B2c, A3c, B3c, B4c, A31, B31, B41,
 ImageSize → 650, PlotRange -> All, BaseStyle → {FontSize → 15},
 PlotLabel → "", Axes → False, Frame → {{True, False}, {True, False}},
 FrameLabel → {{"Log (Im/If)", None}, {"Stress", None}},
 LabelStyle -> Directive[Black]]
```

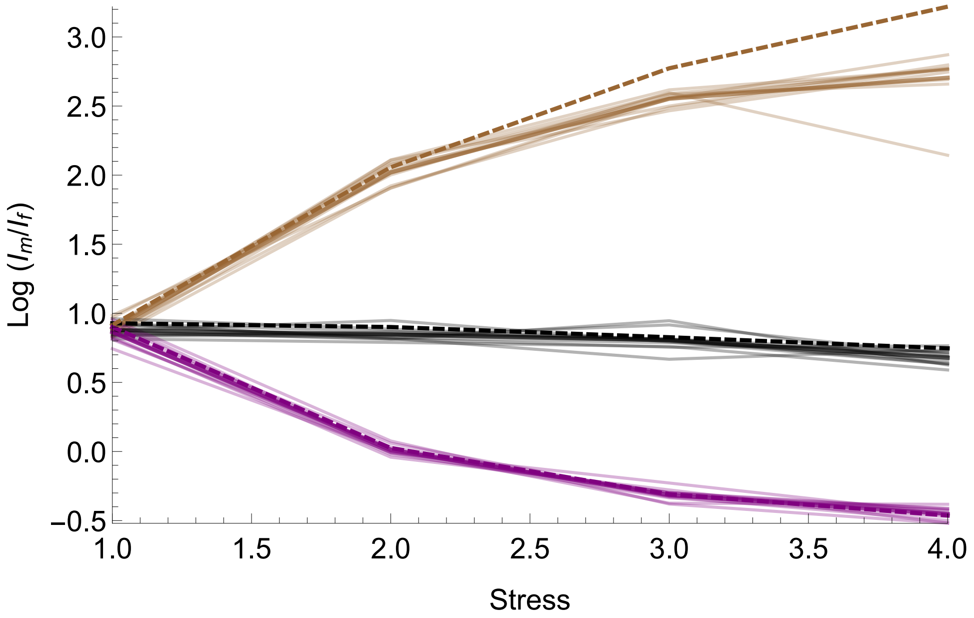

IV. Equal competition in males and females, with or without gifts and harm

4th set of assumptions:

- males contribute little to fecundity.

-No interaction at first

-Slight environmental stress, with no sex specificity.

-Competition equal in males and females.

Question: how does sex specific change in variance in condition affects the opp. for selection?

```
sdf = 1/10;
sdm = 1/10;
popsize = 1000;
imf = 0;
mc = 1/100;
gf = 2;
gm = 2;
```

```
DatF = Table[Table[Datt = Johanna[sdchange, sdchange, popsize, imf, mc, gf, gm];
    {sdchange, Datt[[1]]}, {sdchange, {0.1, 0.2, 0.3, 0.4}}], {num, 0, 10}];
DatM = Table[Table[Datt = Johanna[sdchange, sdchange, popsize, imf, mc, gf, gm];
    {sdchange, Datt[[2]]}, {sdchange, {0.1, 0.2, 0.3, 0.4}}], {num, 0, 10}];
DatBF = Table[Table[Datt = Johanna[sdchange, sdm, popsize, imf, mc, gf, gm];
    {sdchange, Datt[[1]]}, {sdchange, {0.1, 0.2, 0.3, 0.4}}], {num, 0, 10}];
DatBM = Table[Table[Datt = Johanna[sdchange, sdm, popsize, imf, mc, gf, gm];
    {sdchange, Datt[[2]]}, {sdchange, {0.1, 0.2, 0.3, 0.4}}], {num, 0, 10}];
DatCF = Table[Table[Datt = Johanna[sdf, sdchange, popsize, imf, mc, gf, gm];
    {sdchange, Datt[[1]]}, {sdchange, {0.1, 0.2, 0.3, 0.4}}], {num, 0, 10}];
DatCM = Table[Table[Datt = Johanna[sdf, sdchange, popsize, imf, mc, gf, gm];
    {sdchange, Datt[[2]]}, {sdchange, {0.1, 0.2, 0.3, 0.4}}], {num, 0, 10}];

AF = ListLinePlot[DatF, PlotRange → All, PlotStyle → Directive[Black, Opacity[0.3]]];
AFmean2 = ListLinePlot[Mean[DatF], PlotRange → All,
   PlotMarkers → {"◆", 30}, PlotStyle → Directive[Black]];
AM = ListLinePlot[DatM, PlotRange → All, PlotStyle → Directive[Black, Opacity[0.3]]];
AMmean2 = ListLinePlot[Mean[DatM], PlotRange → All,
   PlotMarkers → {"●", 30}, PlotStyle → Directive[Black]];
ABF = ListLinePlot[DatBF, PlotRange → All, PlotStyle → Directive[Purple, Opacity[0.3]]];
ABFmean2 = ListLinePlot[Mean[DatBF], PlotRange → All,
   PlotMarkers → {"◆", 30}, PlotStyle → Directive[Purple]];
ABM = ListLinePlot[DatBM, PlotRange → All, PlotStyle → Directive[Purple, Opacity[0.3]]];
ABMmean2 = ListLinePlot[Mean[DatBM], PlotRange → All,
   PlotMarkers → {"●", 30}, PlotStyle → Directive[Purple]];
ACF = ListLinePlot[DatCF, PlotRange → All, PlotStyle → Directive[Brown, Opacity[0.3]]];
ACFmean2 = ListLinePlot[Mean[DatCF], PlotRange → All,
   PlotMarkers → {"◆", 30}, PlotStyle → Directive[Brown]];
ACM = ListLinePlot[DatCM, PlotRange → All, PlotStyle → Directive[Brown, Opacity[0.3]]];
ACMmean2 = ListLinePlot[Mean[DatCM], PlotRange → All,
   PlotMarkers → {"●", 30}, PlotStyle → Directive[Brown]];

GraphicsRow[{
  Show[AM, AF, AFmean2, AMmean2, AFmean, AMmean, ImageSize → 450, PlotRange -> All,
   BaseStyle → {FontSize → 15}, PlotLabel → "a. Both sexes are stressed",
   Axes → False, Frame → {{True, False}, {True, False}},
   FrameLabel → {{"Im If", None}, {"Stress", None}}, LabelStyle -> Directive[Black]],
  Show[ABM, ABF, ABMmean2, ABFmean2, ABMmean, ABFmean, ImageSize → 450, PlotRange -> All,
   BaseStyle → {FontSize → 15}, PlotLabel → "b. Females only are stressed",
   Axes → False, Frame → {{True, False}, {True, False}},
   FrameLabel → {{None, None}, {"Stress", None}}, LabelStyle -> Directive[Black]],
  Show[ACM, ACF, ACMmean2, ACFmean2, ACMmean, ACFmean, ImageSize → 450, PlotRange -> All,
   BaseStyle → {FontSize → 15}, PlotLabel → "c. Males only are stressed",
   Axes → False, Frame → {{True, False}, {True, False}}, FrameLabel →
    {{None, None}, {"Stress", None}}, LabelStyle -> Directive[Black]]}, Spacings → 1]
```

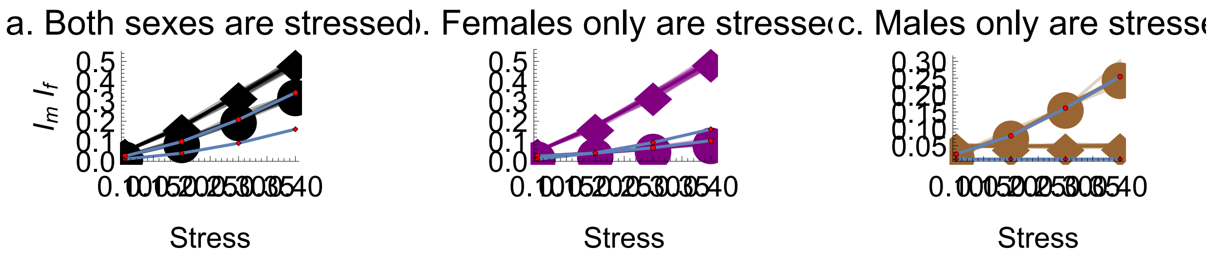

```
DatA = Table[Table[Datat = Johanna[sdchange, sdchange, popsize, imf, mc, gf, gm];
    Log[Datat[[2]] / Datat[[1]]], {sdchange, {0.1, 0.2, 0.3, 0.4}}], {num, 0, 10}];
DatB2 = Table[Table[Datat = Johanna[sdchange, sdm, popsize, imf, mc, gf, gm];
    Log[Datat[[2]] / Datat[[1]]], {sdchange, {0.1, 0.2, 0.3, 0.4}}], {num, 0, 10}];
DatB = Table[Table[Datat = Johanna[sdf, sdchange, popsize, imf, mc, gf, gm];
    Log[Datat[[2]] / Datat[[1]]], {sdchange, {0.1, 0.2, 0.3, 0.4}}], {num, 0, 10}];
Ac = ListLinePlot[DatA, PlotRange → All, PlotStyle → Directive[Black, Opacity[0.3]]];
Bc = ListLinePlot[DatB,
   PlotRange → {{1, 4}, {Min[0, Min[DatB2, DatA, DatB]], Max[DatB2, DatA, DatB]}},
   PlotStyle → Directive[Brown, Opacity[0.3]]];
B2c = ListLinePlot[DatB2, PlotStyle → Directive[Purple, PlotRange → All, Opacity[0.3]]];
A3c = ListLinePlot[Table[Mean[Transpose[DatA][[ä]]], {ä, 1, 4}],
   PlotStyle → Directive[Black, Thickness[0.005], Opacity[0.7]], PlotRange → All];
B3c = ListLinePlot[Table[Mean[Transpose[DatB][[ä]]], {ä, 1, 4}],
   PlotStyle → Directive[Brown, Thickness[0.005], Opacity[0.7]], PlotRange → All];
B4c = ListLinePlot[Table[Mean[Transpose[DatB2][[ä]]], {ä, 1, 4}],
   PlotStyle → Directive[Purple, Thickness[0.005], Opacity[0.7]], PlotRange → All];
```

```
Show[Bc, Ac, B2c, A3c, B3c, B4c, A31, B31, B41,
 ImageSize → 650, PlotRange -> All, BaseStyle → {FontSize → 15},
 PlotLabel → "", Axes → False, Frame → {{True, False}, {True, False}},
 FrameLabel → {{"Log (Im/If)", None}, {"Stress", None}},
 LabelStyle -> Directive[Black]]
```

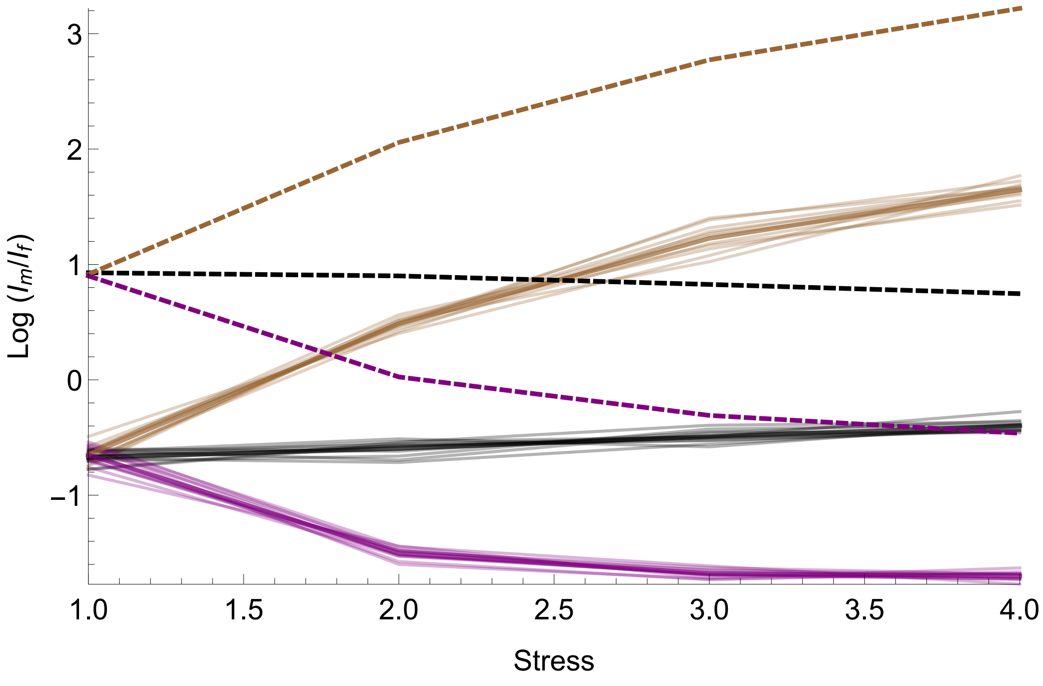

Supplement: Supplementary file 3 — Supplementary Material [file EVO-74-2714-s003.pdf]
